# Transport of a kinesin-cargo pair along microtubules into dendritic spines undergoing synaptic plasticity

**DOI:** 10.1038/ncomms12741

**Published:** 2016-09-23

**Authors:** Derrick P. McVicker, Adam M. Awe, Karl E. Richters, Rebecca L. Wilson, Diana A. Cowdrey, Xindao Hu, Edwin R. Chapman, Erik W. Dent

**Affiliations:** 1 Department of Neuroscience, University of Wisconsin, School of Medicine and Public Health, 1111 Highland Avenue, Madison, Wisconsin 53705, USA; 2 Howard Hughes Medical Institute, University of Wisconsin, School of Medicine and Public Health, 1111 Highland Avenue, Madison, Wisconsin 53705, USA

## Abstract

Synaptic plasticity often involves changes in the structure and composition of dendritic spines. Vesicular cargos and organelles enter spines either by exocytosing in the dendrite shaft and diffusing into spines or through a kinesin to myosin hand-off at the base of spines. Here we present evidence for microtubule (MT)-based targeting of a specific motor/cargo pair directly into hippocampal dendritic spines. During transient MT polymerization into spines, the kinesin KIF1A and an associated cargo, synaptotagmin-IV (syt-IV), are trafficked in unison along MTs into spines. This trafficking into selected spines is activity-dependent and results in exocytosis of syt-IV-containing vesicles in the spine head. Surprisingly, knockdown of KIF1A causes frequent fusion of syt-IV-containing vesicles throughout the dendritic shaft and diffusion into spines. Taken together, these findings suggest a mechanism for targeting dendritic cargo directly into spines during synaptic plasticity and indicate that MT-bound kinesins prevent unregulated fusion by sequestering vesicular cargo to MTs.

## Abstract (web summary)

Transport of cargo into dendritic spines is required for synaptic plasticity. McVicker *et al.* describe a method of activity-dependent transport of a kinesin KIF1A and its cargo synaptotagmin-IV along microtubules that are transiently polymerized into dendritic spines.

## Graphical abstract

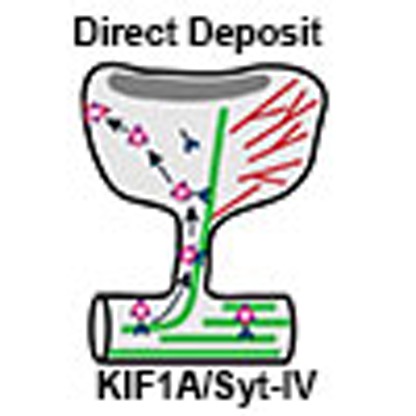

The ability of dendritic spines to alter their molecular composition in response to cellular cues is essential for the strengthening and weakening of connections between neurons during synaptic plasticity. Our current understanding of how cargos are trafficked in and out of spines upon changes in neuronal activity has increased over the years; however, large gaps still remain in the existing models. Most models posit either exocytosis of vesicles in the dendrite shaft followed by diffusion in the plane of the membrane or a myosin-based transport of material from the dendrite shaft into the spine^1^. The actin-based motor proteins myosin-Va (MyoVa) and myosin-Vb (MyoVb) have both been implicated in transporting recycling endosomes and their α-amino-3-hydroxy-5-methyl-4-isoxazolepropionic acid (AMPA) receptor cargo into hippocampal dendritic spines^2^,^3^,^4^. In addition, MyoVa has been shown to traffic endoplasmic reticulum (ER) into dendritic spines of cerebellar Purkinje neurons^5^. These data indicate that there are independent mechanisms for transporting specific cargos into dendritic spines.

One area of growing interest is the contribution of transient microtubule (MT) polymerizations (invasions) into dendritic spines^6^,^7^,^8^. Such MT invasions could provide a direct track for the entry of important cargo into spines. MT invasions have been shown to affect the turnover of the actin-associated protein p140CAP in spines as well as the increase in PSD-95 in spines after brain-derived neurotrophic factor (BDNF) treatment^8^,^9^. However, neither p140Cap nor post synaptic density (PSD)-95 is trafficked into spines along MTs. It is not known whether MTs that polymerize into dendritic spines are capable of kinesin-based transport of cargo directly into dendritic spines. If these MTs are capable of such motor-based transport what type of kinesin(s) are used and what is their cargo? Moreover, what is the fate of the cargo entering along these MTs?

Kinesin family members are responsible for the transport and delivery of unique cargos along MTs. For example, the kinesin-1 family member KIF5B transports mitochondria^10^,^11^, the kinesin-2 family member KIF17 transports specific NMDA receptor subunits^12^,^13^ and the kinesin-3 family member KIF1A transports dense-core vesicles^14^. Generally, it is thought that kinesins and dynein move vesicular cargos throughout dendrites along MTs, where they are released at a specific spine and either exocytose at the base of a particular spine or are transferred into the spine by actomyosin-based transport^13^,^15^. Although it is clear that some materials are transported into spines by myosin, others may move directly into the spine via kinesin-based transport. Transient MT invasions into spines can last on the order of minutes^7^, and the frequency and duration of these invasions can be increased through neuronal activity^7^,^9^. Given that MT-based motors can travel at speeds in excess of 1 μm s^—1^ (refs 16, 17), we hypothesized that, during these transient MT invasions, kinesin motors could transport and deliver cargo directly into the spine head. An attractive candidate motor to transport cargo into spines is the kinesin-3 motor KIF1A. KIF1A has been shown to move cargo in both axons and dendrites^14^,^18^, and has been implicated in synaptogenesis, as well as learning and memory in mice^19^. Several unique cargos have been identified for KIF1A, including synaptic vesicle precursors^20^, and indirect results from immunoprecipitation assays indicate that KIF1A may also be involved in transporting Rab3 and synaptotagmin family members^20^,^21^. Specifically, synaptotagmin-IV (syt-IV) immunoprecipitates with KIF1A (ref. 21), regulates synaptic function and long-term potentiation (LTP), and is a negative regulator of BDNF-induced exocytosis in neurons^22^. Therefore, we determined whether KIF1A and syt-IV act as a motor–cargo pair that is transported along newly polymerized MTs directly into spines.

## Results

### Colocalization of KIF1A and syt-IV in dendrites and spines

**Figure 1:**
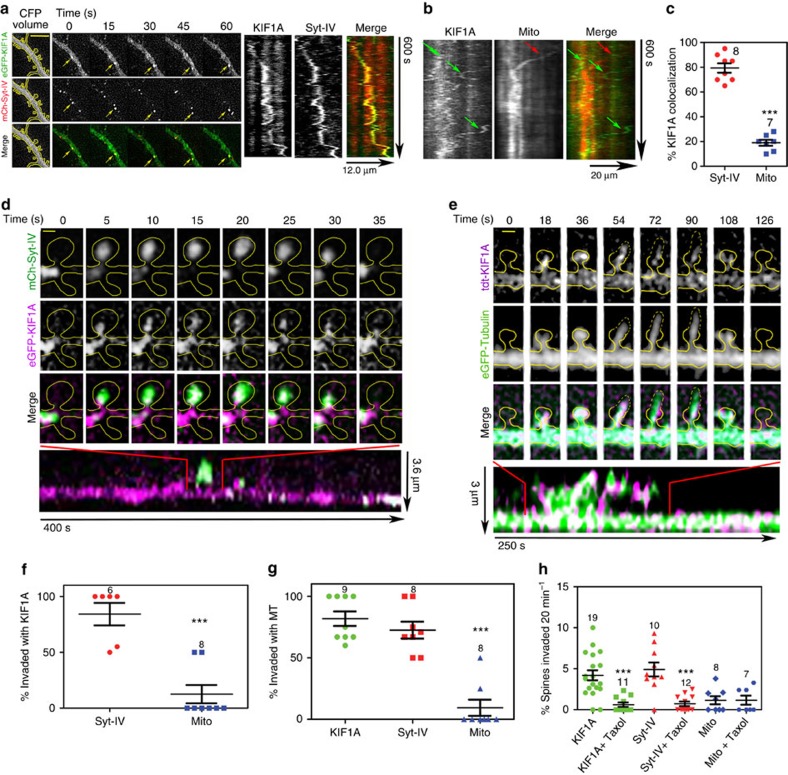
KIF1A dynamically colocalizes with syt-IV and invades dendritic spines along microtubules. (**a**) eGFP-KIF1A colocalizes with mCherry-syt-IV in the dendritic shaft and moves together over time as detected in time-lapse images and in kymographs (scale bar, 5 μm). (**b**) Kymographs demonstrating that KIF1A (green arrows) does not colocalize with mitochondria (red arrow) in the dendritic shaft. (**c**) KIF1A colocalizes with syt-IV significantly more (79.4±3.8%) than with mitochondria (19.0±2.4%) in the dendritic shaft (*P*=0.0009, *n*=8, 7). (**d**) mCherry-syt-IV (green) invades dendritic spines (outlined in yellow from cyan fluorescent protein (CFP) volume fill) simultaneously with eGFP-KIF1A (magenta) for 30 s before both molecules disappear together (scale bar, 1 μm). (**e**) tdTomato-KIF1A (magenta) invades a dendritic spine (outlined in yellow) during a MT invasion (eGFP-tubulin, green; scale bar, 1 μm). (**f**) Syt-IV invades dendritic spines simultaneously with KIF1A (84.2±10.0%, *n*=6) of the time and significantly more than with mitochondria (12.5±8.2%, *P*=0.0032, *n*=8). (**g**) KIF1A and syt-IV invade dendritic spines 81.8±5.9% (*n*=9) and 72.5±6.8% (*n*=8) of the time with MTs, respectively, while mitochondria invade with MTs a significantly lower percentage of time (9.4±6.6, *P*=0.0003, *n*=8). (**h**) Low dose (10 nM) application of Taxol significantly reduces the percentage of spines invaded over a 20 min time period by both KIF1A (from 4.2±0.6 to 0.6±0.3%, *P*=0.0002, *n*=19, 11)) and syt-IV (from 4.9±0.9 to 0.73±0.28%, *P*=0.0001, *n*=10, 12), but not mitochondria (from 1.1±0.5 to 1.1±0.6, *P*=0.9999, *n*=8, 7). Asterisks indicate significance relative to controls (*P<0.05, **P<0.01, ***P<0.001). Numbers above scatter plots indicate number of cells analysed per condition, from at least three replicate experiments. For **c**,**f**, a Student's *t*-test was used to determine significance. For **g**,**h**, a one-way analysis of variance (ANOVA) with Tukey's *post hoc* test was used. All graphs show mean±s.e.m.

Given that KIF1A and syt-IV are both transported along dendrites, are important in synaptic plasticity and syt-IV has been implicated as a KIF1A cargo, we coexpressed both mCherry-syt-IV and eGFP-KIF1A in rat primary hippocampal neurons and imaged the dynamic localization of these proteins in living neurons. We focused on neurons that had relatively low transfection levels, appeared morphologically normal and had active MT, motor and cargo dynamics. KIF1A puncta colocalized with syt-IV puncta on average 79.4±3.8% of the time over a 20-min period and they both moved together in the dendritic shaft (Fig. 1a,c). To ensure that this was a specific interaction and not a consequence of syt-IV overexpression, we also examined the colocalization of KIF1A with mitochondria (labelled with DsRed2-Mito), which are known cargoes of KIF5 (refs 10, 11), and have not been reported to be transported by KIF1A (refs 23, 24). Although we acknowledge that mitochondria may have different trafficking kinetics than syt-IV-containing vesicles, we observed that KIF1A colocalized with mitochondria on average only 19.0±2.4% of the time over a 20-min period (Fig. 1b,c), which is inconsistent with KIF1A being a motor for mitochondria and suggests that the dynamic colocalization of KIF1A and syt-IV is not a consequence of overexpression. Interestingly, we noted that there was compartmental specificity with regard to KIF1A and syt-IV colocalization and trafficking. KIF1A moved anterogradely in axons; however, syt-IV moved primarily retrogradely (Supplementary Fig. 1), and unlike in dendrites, little colocalization between KIF1A and syt-IV puncta was observed. These data indicate that KIF1A is a dendrite-specific carrier of syt-IV, and in axons syt-IV is primarily transported retrogradely, in a manner similar to BDNF-containing vesicles^22^.

**Figure 2:**
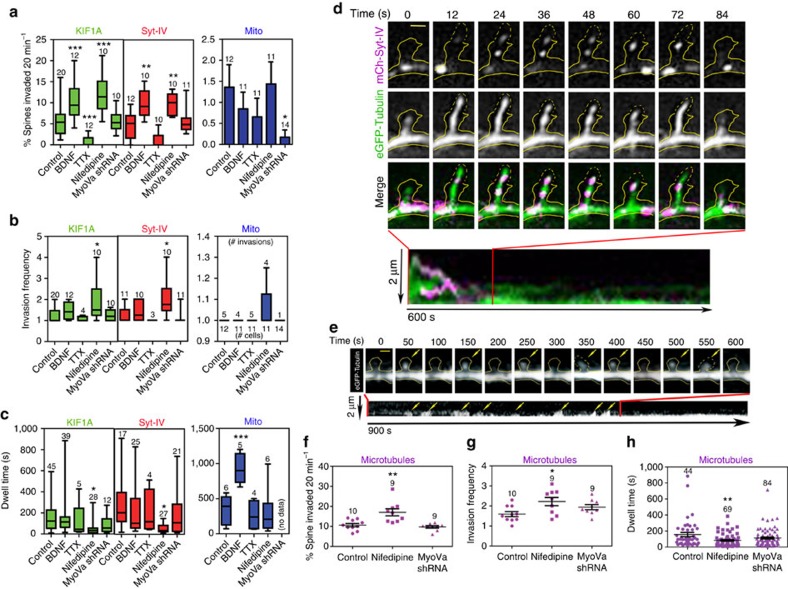
KIF1A and syt-IV invasions into dendritic spines are activity-dependent. (**a**) The percentage of spines invaded by KIF1A and syt-IV over a 20 min period increased significantly with a 20 min BDNF treatment (KIF1A *P*=0.00045, Syt-IV *P*=0.00485) and 24-h nifedipine treatment (KIF1A *P*=0.0001, Syt-IV *P*=0.0054). With a 20 min application of TTX the percentage of spines invaded by KIF1A decreased significantly (*P*=0.0006). Unlike KIF1A and syt-IV, the percentage of spines invaded by mitochondria with these treatments was not statistically significant from control neurons (BDNF, TTX and nifedipine all *P*>0.9). Knockdown of myoVa only significantly reduced the percentage of spines invaded by mitochondria (*P*=0.04). (**b**) Nifedipine increased the invasion frequency of both KIF1A (*P*=0.0389) and syt-IV (*P*=0.0178). Other treatments were without effect. Because there were very few invasions of mitochondria, both the number of cells and the number of invasions are indicated on the Mito graph. (**c**) Twenty-four hour nifedipine treatment significantly reduced the dwell times of both KIF1A (*P*=0.0084) and syt-IV (*P*=0.0044). BDNF treatment significantly increased the dwell time of mitochondria in spines (*P*=0.0049). (**d**) After a 20 min bath treatment of 50 ng ml^−1^ BDNF two puncta of mCherry-syt-IV (magenta) invaded a dendritic spine (outlined in yellow) along an invading MT (green; scale bar, 1 μm). (**e**) MTs frequently invaded dendritic spines (yellow arrows) after 24 h of nifedipine treatment (scale bar, 1 μm). (**f**–**h**) After a 24 h nifedipine treatment the percentage of spines invaded by MTs over 20 min (*P*=0.0016) and the MT invasion frequency (*P*=0.0484) increased significantly, while the dwell time of MTs decreased significantly (*P*=0.0045), as compared with control neurons and cells expressing shRNA against myoVa. Asterisks indicate significance relative to controls (*P<0.05, **P<0.01, ***P<0.001). Numbers above box and whisker or scatter plots indicate number of cells analysed per condition (graphs **a**,**f**,**g**) or number of spine invasions (graphs **b**,**c**,**h**), from at least three replicate experiments. For all statistics, a one-way ANOVA with Tukey's *post hoc* test was used. All graphs show mean±s.e.m.

To determine whether KIF1A and syt-IV moved together into dendritic spines, we coexpressed eGFP-KIF1A and mCherry-syt-IV and tracked their entry into spines (Fig. 1d). We noted that 84.17±10.0% of syt-IV invasions were accompanied by a corresponding KIF1A invasion, but KIF1A only invaded with mitochondria 12.5±8.2% of the time (Fig. 1f). When tdTomato-KIF1A or mCherry-syt-IV were coexpressed with eGFP-tubulin, we observed that both the motor (Fig. 1e) and the cargo (Fig. 2d) entered spines coincident with transient MT polymerizations (invasions) 81.8±5.9% and 72.5±6.8% of the time, respectively (Fig. 1g). However, mitochondria entered with MTs only 9.4±6.6% of the time (Fig. 1g). Moreover, KIF1A and syt-IV invaded a similar percentage of spines over a 20-min time interval (Fig. 1h), while mitochondria entered significantly fewer spines (Fig. 1h). This percentage of KIF1A and syt-IV co-translocation is probably an underestimate because only a few motors are needed to transport cargo. Thus, it is possible that the syt-IV entering spines without observed KIF1A may correspond to an undetectable level of enhanced green fluorescent protein (eGFP)–KIF1A or may be moving by unlabelled, endogenous KIF1A. After spine invasions, a variety of behaviours exhibited by the labelled puncta were observed over a 20-min imaging regimen. KIF1A remained in spines after invasion 16.3±6.4% of the time, disappeared 24.2±5.6% of the time and exited spines after invasion 47.4±8.2% of the time, while 11.9±4.8% of KIF1A puncta were present in the spine the entire duration of the time lapse (Supplementary Fig. 2a). Consistently, syt-IV puncta exhibited similar behaviours (Supplementary Fig. 2a). In addition, the mean square displacement (MSD) analysis of syt-IV puncta moving in the dendritic shaft and entering spines indicated that the majority of these events were directed motion, and not randomly diffusing events, indicating that the cargo was processively moved down the dendritic shaft and into spines and was not diffusing in the cytoplasm or plasma membrane (Supplementary Fig. 3a,b).

To determine whether MT dynamics were necessary for KIF1A and syt-IV invasions of spines, we added 10 nM Taxol to cultures during time-lapse imaging. Previously, we showed that low doses of Taxol significantly reduced the number of MT invasions into spines, without disrupting levels of MTs in the dendritic shaft^9^. Taxol reduced the number of KIF1A and syt-IV invasions by over fivefold (Fig. 1h). However, invasions of mitochondria were unaffected by the addition of Taxol, and remained at a low level (Fig. 1h). Interestingly, the majority of KIF1A, syt-IV and MT invasions penetrated deep within the spine head, presumably into the PSD, and occasionally caused the spine to transiently change shape (Figs 1e and 2d). Mitochondria, however, entered the neck of the spine and only penetrated slightly into the spine head, rarely altering spine morphology (Supplementary Fig. 2e).

### Activity-dependent spine entry of KIF1A and syt-IV

Neuronal activity can have a profound effect on the number of spines invaded by MTs, MT invasion frequencies and dwell times^7^,^9^,^25^,^26^. For example, BDNF secretion accompanies increases in synaptic activity^27^, and bath application of BDNF to cell cultures dramatically increases the dwell time of MT invasions in spines^9^. Conversely, neurons exposed to the sodium channel blocker tetrodotoxin (TTX) for a short time trended towards a reduced number of spines containing MTs^7^. Thus, we expected that the behaviour of KIF1A and syt-IV moving into spines along MTs would also be altered with changes in synaptic activity.

Indeed, with application of BDNF the percentage of spines invaded increased significantly for KIF1A and syt-IV (10.5±1.4%, *P*=0.00045, *n*=12 for KIF1A and 9.8±1.0%, *P*=0.00485, *n*=10 for syt-IV) as compared with control neurons (5.5±0.9%, *n*=20 for KIF1A and 4.8±0.91%, *n*=12 for syt-IV; Fig. 2a,b). When mitochondria were observed with the same treatments, BDNF had no observable effect on the percentage (0.9±0.4%, *P*=0.9999, *n*=11) or frequency (1.0±0.0, *P*=0.9999, *n*=4) of mitochondrial invasions as compared with control neurons (1.4±0.5%, *n*=12; 1.0±0.0, *n*=4; Fig. 2a,b). However, the dwell times of mitochondrial invasions were significantly increased by BDNF (from 348.0±83.1 s for control cells to 929.2±95.6 s with the addition of BDNF, *P*=0.0049, *n*=5), while KIF1A and syt-IV dwell times were unaffected (151.5±25.6 s, *P*=0.9942, *n*=39 for KIF1A and 130.4±27.8 s, *P*=0.9998, *n*=25 for syt-IV) as compared with controls (160.2±22.3 s, *n*=45 for KIF1A and 171.1±34.1 s, *n*=17 for syt-IV; Fig. 2c). There was an increase in the number of syt-IV puncta, but not KIF1A puncta, that either remained in the spines after invasion or were in the spine the entire duration of the 20-min time lapse (Supplementary Fig. 2b). KIF1A puncta primarily exited the spine or disappeared upon BDNF treatment (Supplementary Fig. 2b), leaving the syt-IV puncta behind. These data indicate that the kinesin either unloads the cargo in the spine head and then exits the spine or diffuses into the dendrite.

In contrast to BDNF addition, KIF1A, syt-IV and mitochondria experienced a marked decrease in the percentage of spines invaded with a 20-min exposure to TTX (0.8±0.4%, *P*=0.0006, *n*=12 for KIF1A, 1.0±0.6%, *P*=0.1411, *n*=10 for syt-IV, and 0.7±0.4%, *P*=0.9999, *n*=11 for mitochondria; Fig. 2a). TTX had no apparent effect on the invasion frequencies (1.1±0.1 for KIF1A, 1.0±0.0 for syt-IV and mitochondria, *P*=0.9999, *n*=4, 3, 5) or dwell times (116.4±78.0 s for KIF1A, *P*=0.9975, *n*=5, 120.0±64.8 s for syt-IV, *P*=0.9999, *n*=4 and 261.0±107.1 s for mitochondria, *P*=0.9997, *n*=4) of the motor or cargos observed (Fig. 2b,c). However, KIF1A and syt-IV puncta tended to primarily exit the spines and no puncta were observed to disappear (Supplementary Fig. 2c).

### Homeostatic plasticity affects KIF1A and syt-IV spine entry

Chronic blockade of L-type calcium channels with the drug nifedipine strongly activates the retinoic acid synthesis pathway, leading to a form of homeostatic plasticity and an increase in AMPA receptor function^28^. On the basis of these data, we predicted that a long-term nifedipine treatment would alter MT dynamics, leading to an increase in kinesin-mediated trafficking into spines. After chronic (24 h) nifedipine treatment, both the percentage of spines invaded by MTs (17.0±1.8%, *P*=0.0016, *n*=9) and the invasion frequency (2.2±0.2, *P*=0.0484, *n*=9) of MTs were significantly increased as compared with controls (10.6±0.8% , *n*=10 for per cent invaded and 1.6±0.6, *n*=10 for frequency; Fig. 2f,g), while MT dwell times significantly decreased from 156.1±26.7 s, *n*=44 in control cells to 83.7±9.1 s for nifedipine treatment (*P*=0.0045, *n*=69 invasions; Fig. 2h). The increase in MT invasion rates and frequencies into spines correlated with an increased number and frequency of invasions of both KIF1A and syt-IV (12.1±1.5%, *P*=0.0001, *n*=10 for KIF1A invasions and 9.8±0.8%, *P*=0.0054, *n*=10 for syt-IV; Fig. 2a,b). Similar to the decrease in MT dwell times, the dwell times of both KIF1A and syt-IV were significantly reduced from control levels of 160.2±22.3 s (*n*=45 invasions) for KIF1A and 171.1±34.1 s (*n*=17 invasions) for syt-IV to 44.7±10.80 s (*P*=0.0084, *n*=28 invasions) and 34.2±4.8 s (*P*=0.0044, *n*=27 invasions), respectively (Fig. 2c). Although at basal levels KIF1A and syt-IV have similar dwell times to MTs, they are not always observed exiting the spine with the MT. While this does occur, KIF1A and syt-IV are also observed to exit the spine while the MT is still in the spine, to disappear or to remain in the spine after the MT depolymerizes out of the spine (Supplementary Fig. 2d). KIF1A and syt-IV puncta were also observed in spines before MT invasions, and only exited after a MT invaded (Supplementary Fig. 4). Interestingly, the dwell times of both syt-IV and KIF1A were significantly shorter than MT dwell times after nifedipine treatment (Fig. 2c,h), and KIF1A and syt-IV puncta were often observed exiting the spine soon after their invasion.

The invasion frequency of mitochondria after chronic nifedipine treatment trended towards increasing (1.4±0.5%, *P*=0.9999, *n*=4), but was not significantly different from the controls (Fig. 2b), while the dwell times were unaffected (312.0±105.1 s, *P*=0.9999, *n*=6; Fig. 2c). Moreover, MTs, KIF1A and syt-IV invasions did not appear to be coordinated with mitochondrial invasions. Mitochondria targeted many of the same spines as the MTs and motor–cargo pairs, but did not invade simultaneously with these molecules, suggesting they were all targeting-active spines, but through separate mechanisms.

To determine the mechanism by which mitochondria were entering spines, we knocked down MyoVa because this motor was previously shown to be responsible for the entry of both ER^5^ and recycling endosomes^2^,^4^ into spines. These experiments were also performed to determine whether MyoVa short hairpin RNA (shRNA) had any effect on KIF1A and syt-IV invasions. Importantly, knockdown of MyoVa (Supplementary Fig. 5) did not affect the percentage of spines invaded (9.7±0.6%, *P*=0.8480, *n*=9), the invasion frequency (1.9±0.1, *P*=0.2617, *n*=9) or the dwell time (112.7±11.5 s, *P*=0.1166, *n*=84 invasions) of MTs in spines (Fig. 2f–h). Moreover, knockdown of MyoVa had no effect on the percentage of spines invaded (5.5±0.7%, *P*=0.9999, *n*=10; 5.8±1.0%, *P*=0.9999, *n*=11), the invasion frequency (1.3±0.1, *P*=0.9999, *n*=10; 1.1±0.3, *P*=0.9999, *n*=11) or the dwell time (92.3±12.9 s, *P*=0.9961, *n*=12; 118.1±28.5 s, *P*=0.9975, *n*=21) of either KIF1A or syt-IV (Fig. 2a–c), respectively. However, it did significantly decrease the percentage of spines invaded (0.2±0.6%, *P*=0.04, *n*=14) by mitochondria (Fig. 2a). These data demonstrate that MyoVa is not functioning to traffic KIF1A or syt-IV into spines, while it suggests that mitochondria enter dendritic spines through a MyoVa-based mechanism, similar to ER and recycling endosomes.

### Syt-IV-containing vesicles undergo membrane fusion in spines

**Figure 3:**
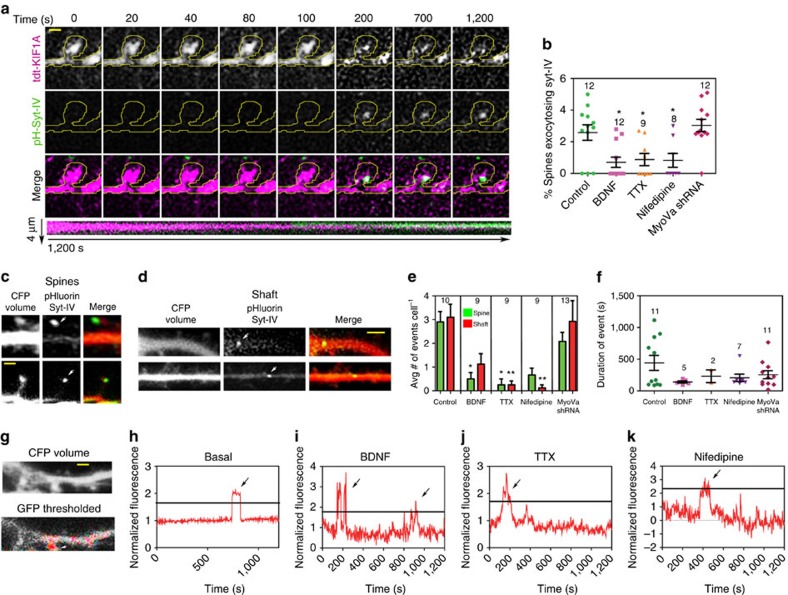
Syt-IV cargo is inserted into the plasma membrane during KIF1A invasions. (**a**) A tdTomato-KIF1A puncta (magenta) is in a dendritic spine (outlined in yellow) at the beginning of the time lapse, and the syt-IV cargo is inserted into the plasma membrane as demonstrated by an increase in the fluorescence of pHluorin-syt-IV signal (green) at 200 s (scale bar, 1 μm). (**b**) BDNF (*P*=0.0110), TTX (*P*=0.0453) and nifedipine (*P*=0.0466) treatments all significantly decreased the percentage of spines exocytosing syt-IV compared with controls. (**c**) Two examples of pHluorin fluorescence in spine heads (scale bar, 1 μm) and (**d**) in the dendritic shaft during an exocytotic event (scale bar, 5 μm). (**e**) The average number of exocytosis events per neuron in the spine (2.90±0.43 per event, *n*=10) and shaft (3.1±0.6 per events, *n*=10) over a 20 min imaging period were reduced with the application of BDNF (0.5±0.3 per event for spines, *P*=0.0316 and 1.3±0.4 per event for the shaft, *P*=0.1169, *n*=9), TTX (0.3±0.3 per event for spines, *P*=0.0200 and 0.3±0.2 per event for the shaft, *P*=0.0086), *n*=9) and nifedipine (0.7±0.3 per event for spines, *P*=0.0738 and 0.1±0.1 per event for the shaft, *P*=0.0049, *n*=9). MyoVa shRNA had no significant effect on exocytosis events in the spines (2.7±0.8 per event) or in the shaft (2.9±0.9 per event, *n*=13). (**f**) Although BDNF, TTX and nifedipine treatments reduced the duration of the observed fluorescent events, statistically there was no difference between these treatments (441.9±118.4 s for Control, *n*=11; 141.0±17.2 s for BDNF, *P*=0.2402, *n*=5; 231.5±99.5 s for TTX, *P*=0.8364, *n*=2; 207.1±57.6 s for nifedipine, *P*=0.3704, *n*=7; and 255.5±64.2 for MyoVa shRNA, *P*=0.4751, *n*=11). (**g**) Intensity data were analysed by thresholding the GFP channel to 10 s.d.'s above the background and measuring the fluorescence intensity over time (scale bar, 5 μm). (**h**–**k**) Only measurements above the threshold were counted as exocytotic events as indicated by the black bars. Asterisks indicate significance relative to controls (*P<0.05, **P<0.01). Numbers above scatter plots indicate number of cells analysed per condition (graphs **b**,**e**) or number of exocytosis events (graph **f**), from at least three replicate experiments. For all statistics, a one-way ANOVA with Tukey's *post hoc* test was used. All graphs show mean±s.e.m.

To determine whether the cargo (presumptive vesicles containing syt-IV) that is being delivered to the spine head during transient MT invasions actually fuses with the spine plasma membrane, neurons were co-transfected with pHluorin-syt-IV, tdTomato-KIF1A and pmTurquoise, as a volume marker. The pHluorin construct is a pH-sensitive fluorophore that is quenched inside acidic vesicles, but exhibits a marked increase in fluorescence upon exocytosis^29^. The pHluorin-syt-IV construct has previously exhibited a prolonged increase in fluorescence upon delivery of syt-IV to the plasma membrane of dendrites^30^. We found that the majority of exocytotic events coincided with KIF1A invasions of spines when KIF1A had longer dwell times or was in the spine for the majority of the time lapse (Fig. 3a). Rarely was exocytosis observed when KIF1A quickly entered and exited the spine. Moreover, we discovered that 2.6±0.5% (*n*=12) of spines exhibited syt-IV exocytosis under basal conditions (Fig. 3b), and the mean percentage of syt-IV exocytosing coincided with kinesin invasions 61.5±12.9% (*n*=10 cells) of the time over a 20-min period. At basal levels, pHluorin-syt-IV exocytosis was observed in both dendritic spines and shafts (Fig. 3c,d). We defined an exocytic event as at least a 10 s.d. increase in fluorescence in images where the GFP signal was thresholded (Fig. 3g,h). Using this criterion (see details in Methods), we demonstrate that there were 2.9±0.4 events in the spine and 3.1±0.6 events in the dendrite shaft (Fig. 3e). The average duration of each event was 441.9±118.4 s, although the duration varied extensively (Fig. 3f). Together, these data indicate that syt-IV-containing vesicles exocytose both in spines, coincident with kinesin invasions, and shafts.

To determine whether exocytosis of syt-IV scaled with activity, we treated neurons with BDNF, TTX and nifedipine, as above. Although BDNF treatment increased the percentage of spines invaded and the invasion frequency of both KIF1A and syt-IV (Fig. 2a,b), an overall reduction in the number of exocytotic syt-IV-containing vesicles was observed (0.7±0.3%, *P*=0.011, *n*=12) after these treatments (Fig. 3b). In addition, no long-lasting fluorescent events were observed after BDNF treatment (Fig. 3f,i). Thus far, syt-IV is the only synaptotagmin family member that has been shown to inhibit exocytosis, and is a negative regulator of BDNF exocytosis^22^. It may seem counterintuitive that BDNF would increase the dwell times of MTs^9^, and the invasion number and frequency of KIF1A and syt-IV (Fig. 2a,b), yet at the same time inhibit the exocytosis of the syt-IV (Fig. 3b). However, KIF1A transports many different cargos, and this increase in trafficking after BDNF addition may be a general mechanism for delivering various cargos to the spine head. The fact that BDNF treatment increased the number of syt-IV puncta that remained in the spine and the number of syt-IV puncta in the spine during the entire time lapse (Supplementary Fig. 2b), but not the corresponding KIF1A puncta, may indicate that the vesicle–cargo pair is being released in the spine head, but has a low propensity of fusing with the plasma membrane. These results are consistent with the function of syt-IV as a negative regulator of exocytosis^22^, and the likelihood that addition of exogenous BDNF would decrease the need for the postsynaptic neuron to release endogenous BDNF through exocytosis.

Similar to BDNF addition, a decrease in the percentage of spines showing exocytotic events was observed with acute applications of the sodium channel blocker TTX (0.9±0.4%, *P*=0.0453, *n*=9) or the chronic application of the L-type calcium blocker nifedipine (0.8±0.4%, *P*=0.0466, *n*=8; Fig. 3b) and the events that were observed were generally short-lived fluorescence increases (Fig. 3f,j,k). Although this decrease in exocytosis of syt-IV-containing vesicles was expected with the 10 min TTX application, because a general decrease in KIF1A and syt-IV invasions was observed, the chronic nifedipine treatment significantly increased the number and frequency of both KIF1A and syt-IV invasions (Fig. 2a,b). Thus, we expected to see an increase in syt-IV exocytosis. As mentioned, at basal levels, the exocytosis of syt-IV primarily coincided with prolonged KIF1A dwell times in spines. Because chronic nifedipine treatment greatly reduced the dwell times of both KIF1A and syt-IV (Fig. 2c) and caused these complexes to exit the spine (Supplementary Fig. 2d), it may indicate that, although there was a general increase in material transported into spines, this particular motor–cargo pair cannot be effectively unloaded and/or is rapidly removed from the spine via dynein-mediated transport in the absence of calcium.

To determine whether exocytosis of syt-IV-containing vesicles was regulated by an actomyosin-based mechanism, we knocked down MyoVa with shRNA (Supplementary Fig. 5) and monitored pHlourin-syt-IV fluorescence. MyoVa knockdown had no effect on the percentage of spines exocytosing syt-IV (3.0±0.4%, *P*=0.9235, *n*=12; Fig. 3b), the number of exocytotic events in the dendrite shaft or spine (Fig. 3e) or the duration of fluorescent events (Fig. 3f). Thus, although we cannot exclude all myosin family members, it is unlikely that entry of syt-IV-containing vesicles and exocytosis in spines is dependent on MyoVa, while mitochondria appear to be sensitive to MyoVa knockdown (Fig. 2a–c).

### Knockdown of KIF1A increases syt-IV entry into spines

**Figure 4:**
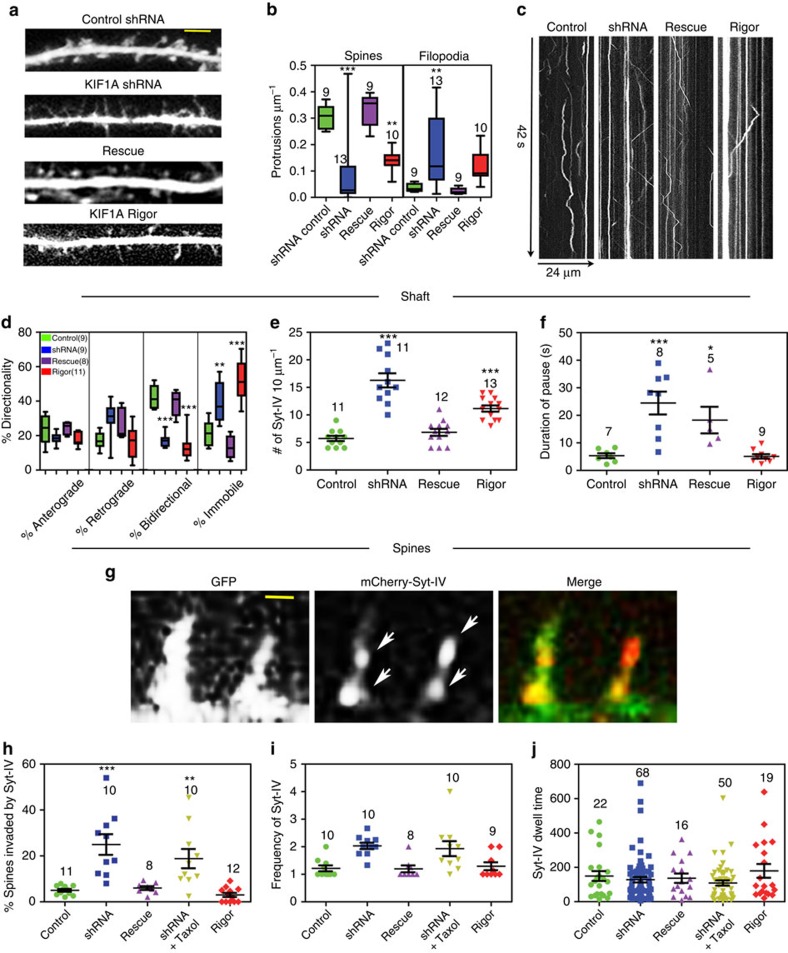
shRNA-mediated knockdown of KIF1A and the expression of the T312M KIF1A rigor mutant caused spine defects and aberrant syt-IV trafficking. (**a**) Representative images of dendrites showing spines and filopodia (scale bar, 5 μm). (**b**) Expression of KIF1A shRNA (*P*=0.0001) or the KIF1A rigor mutant (*P*=0.0014) decreased the spine density and KIF1A shRNA increased the filopodial density (*P*=0.0055) compared with control cells. (**c**) Representative kymographs over 42 s show movement of syt-IV puncta over time. (**d**) Syt-IV bidirectional movement decreased significantly in neurons expressing either KIF1A shRNA (*P*=0.0001) or KIF1A rigor mutant (*P*=0.0001). However, the percentage of immobile syt-IV puncta increased in neurons expressing KIF1A shRNA (*P*=0.0076) or the KIF1A rigor mutant (*P*=0.0001). (**e**) Expression of KIF1A shRNA (*P*=0.0001) or the KIF1A rigor mutant (*P*=0.0002) led to a significant increase in the number of syt-IV puncta throughout the dendritic arbor. (**f**) Syt-IV puncta in neurons expressing KIF1A shRNA (*P*=0.0004) or KIF1A shRNA and the recue construct (*P*=0.0404) demonstrated an increase in the duration of pausing as compared with control neurons. (**g**,**h**) KIF1A shRNA expression led to a significant increase in the percentage of protrusions invaded by syt-IV (*P*=0.0001) and often times multiple puncta of syt-IV (white arrows) accumulated in protrusions at any given time point (scale bar, 1 μm). The addition of Taxol to cells treated with KIF1A shRNA (*P*=0.0069) was also significantly different from controls but is not significantly different from KIF1A shRNA alone (*P*=0.5346). (**i**,**j**) The frequency of syt-IV invasions and the dwell time of syt-IV puncta were not significantly different in any conditions tested. Asterisks indicate significance relative to controls (*P<0.05, **P<0.01, ***P<0.001). Numbers above box and whisker and scatter plots indicate the number of cells analysed (graphs **b**,**d**,**e**,**h**,**i**) and the number of syt-IV puncta (graphs **f**,**j**) from at least three replicate experiments. For all statistics, a one-way ANOVA with Tukey's *post hoc* test was used. All graphs show mean±s.e.m.

To further characterize the role of KIF1A in both the maintenance of spines and as a syt-IV-trafficking motor in dendrites, we knocked down KIF1A with shRNA (KIF1A-kd). We detected a 54.0±5.7% knockdown of KIF1A via immunocytochemistry in neurons expressing the shRNA construct (Supplementary Fig. 6a,b). Moreover, by western blot of HEK293 cells expressing the eGFP-KIF1A rat construct and shRNA, we detected a 69.8% knockdown (Supplementary Fig. 6c,d). KIF1A-kd neurons exhibited a significant decrease in spine density (0.08±0.04 protrusions μm^−1^, *P*=0.0001, *n*=13) and an increase in filopodia (0.17±0.03 protrusions μm^−1^, *P*=0.0055, *n*=13) compared with control shRNA-expressing neurons (0.31±0.02 protrusions μm^−1^, *n*=9 for spines and 0.04±0.01 protrusions μm^−1^, *n*=9 for filopodia; Fig. 4a,b). When mCherry-syt-IV was expressed in KIF1A-kd neurons, aberrant syt-IV trafficking was observed in the dendrite shaft (Fig. 4c). Syt-IV puncta showed more immobility (38.9±3.8%, *P*=0.0076, *n*=9) and decreased bidirectional movement (17.2±1.2%, *P*=0.0001, *n*=9) compared with puncta in control cells (17.4±4.1% for immobile events and 42.6±2.3% for bidirectional events, *n*=9; Fig. 4d). Although syt-IV puncta were well distributed throughout the dendritic arbor, the puncta paused for a much longer period of time (24.5±4.2 s, *P*=0.0004, *n*=8) compared with control cells (5.7±0.9 s, *n*=7; Fig. 4f), leading to a significant increase in the number of puncta in the dendritic shaft at any given time (16.3±1.3 puncta 10 μm^−1^, *P*=0.0001, *n*=11) as compared with control cells (5.7±0.5 puncta 10 μm^−1^, *n*=11; Fig. 4e). Despite the increase in puncta number, there was not a significant increase in the velocity of syt-IV or the brightness of puncta (Supplementary Fig. 6f,g), indicating that the expression level of syt-IV was maintained in these neurons. In addition, MSD analysis revealed a decrease in the number of syt-IV puncta exhibiting directed transport and an increase in randomly diffusing puncta (Supplementary Fig. 3b). This disruption of syt-IV movement was specific for the KIF1A cargo, as mitochondria were observed to move normally throughout the dendritic shaft (Supplementary Fig. 6e).

To ensure that the observed spine and transport defects were not a result of off-target effects, a shRNA-resistant eGFP-KIF1A mutant that harbours five silent mutations in the region complimentary to the shRNA was coexpressed with mCherry-syt-IV and a pmTurquoise-shRNA plasmid, in an attempt to rescue the knockdown phenotype. The ability of this mutant to resist shRNA-mediated knockdown was confirmed by western blot (Supplementary Fig. 6c,d). Neurons that strongly expressed the KIF1A shRNA-resistant mutant, and had observable KIF1A puncta moving throughout the dendritic arbor, had protrusion morphology similar to control neurons (0.33±0.03 protrusions μm^−1^, *P*=0.9997, *n*=9 for spines and 0.02±0.01 protrusions μm^−1^, *P*=0.9999, *n*=9 for filopodia; Fig. 4a,b) and similar syt-IV transport kinetics in both the dendrite shaft (6.8±0.6 puncta per 10 μm^−1^, *P*=0.7547, *n*=12; Fig. 4d,e) and dendritic spines (6.0±0.8%, *P*=0.9992, *n*=8; Fig. 4h). However, the duration of pause in syt-IV puncta was still slightly elevated in KIF1A-rescued neurons (18.3±4.8 s, *P*=0.0404, *n*=5; Fig. 4f). Taken together, these results indicate that disruptions in syt-IV trafficking were due to the reduced amount of KIF1A in the neurons and were not due to off-target effects of the shRNA.

If KIF1A is indeed the motor that transports syt-IV-containing vesicles into spines, then one would expect a decrease in syt-IV invasions of spines after KIF1A knockdown. Surprisingly, the percentage of spines invaded by syt-IV (26.0±4.5%, *P*=0.0001, *n*=10) and the frequency of syt-IV invasions (2.0±0.1, *P*=0.0068, *n*=10) significantly increased in the KIF1A-kd neurons as compared with controls (5.0±0.6%, *n*=11; 1.2±0.1, *n*=10; Fig. 4h,i). In addition, the percentage of syt-IV puncta that remained in the dendritic protrusions throughout the entire time-lapse increased (Supplementary Fig. 6i), despite the dwell times of syt-IV puncta (128.2±15.8 s, *P*=0.9600, *n*=68 invasions) remaining unchanged (Fig. 4j). In many cases, multiple puncta were observed in a protrusion at any given time (Fig. 4g), which was rarely seen in control neurons. These data could indicate that without the proper motor protein present the syt-IV vesicles enter spines in an unregulated manner.

To determine whether the increase in syt-IV invasions was due directly to altered MT polymerization into spines, we attempted to analyse the percentage and frequency of protrusions entered by MTs using eGFP-tubulin and the pmTurquoise-expressing KIF1A-shRNA plasmid. In neurons expressing shRNA, there appeared to be an excess of free tubulin in the cytoplasm, making the visualization and analysis of MT invasions impossible. Therefore, we expressed tdTomato-EB3, which is a MT +TIP protein that labels the polymerizing ends of MTs in a comet-like appearance^31^. We and others have used EB3 to monitor MT dynamics in dendrites and spine invasions in neurons^8^,^26^. We measured EB3 velocity, track length and lifetimes of comets in the dendrite shaft, as well as the percentage of spines invaded and the frequency of EB3 invasions in spines, but detected no significant differences between control and KIF1A-kd neurons (Supplementary Fig. 7a–f). These data indicate that MT polymerization kinetics appear to be conserved in the KIF1A-kd dendrites and spines.

Conversely, we tested whether inhibiting MT entry into spines with 10 nM Taxol addition affected the marked increase in percentage of spines invaded by syt-IV after KIF1A-kd (Fig. 4h). We reasoned that if MT polymerization into spines was important for syt-IV entry after KIF1A-kd then addition of Taxol should inhibit syt-IV invasions greatly. However, Taxol addition to the KIF1A-kd neurons still resulted in a significant increase in the percentage of spines invaded by syt-IV (18.8±4.2%, *P*=0.0069, *n*=10) and was not significantly different than KIF1A-kd, even though we suppressed MT polymerization into spines (Fig. 4h). The frequency of syt-IV invasions (1.9±0.3, *P*=0.9924, *n*=10) and their dwell time (108.9±15.2 s, *P*=0.9283, *n*=50) was also not significantly different than KIF1A-kd (Fig. 4i,j). These results suggest that MT dynamics are not involved in the increase in syt-IV entry into spines after KIF1A-kd.

As a further test to determine whether any kind of disruption of KIF1A resulted in excessive entry of syt-IV into spines, we transfected a rigor mutant of KIF1A (KIF1A-T312M) into neurons^32^. KIF1A-T312M has a point mutation in the motor domain, compromising its processive activity. Not surprisingly, the rigor mutant decreased the number of spines 0.14±0.01 protrusions μm^−1^, *P*=0.0014, *n*=10) and trended towards an increase in the number of filopodia (0.12±0.02 protrusions μm^−1^, *P*=0.5169, *n*=10; Fig. 4a,b). The KIF1A rigor mutant also increased the percentage of immobile syt-IV puncta (51.9±3.8%, *P*=0.0001, *n*=11), decreased the percentage of bidirectional puncta (13.8±2.3%, *P*=0.0001, *n*=11) and increased the overall number of puncta in the dendrite shaft (11.2±0.6 puncta 10 μm^−1^, *P*=0.00021, *n*=13) in much the same way as KIF1A-shRNA (Fig. 4c–e). However, unlike KIF1A-shRNA, the KIF1A rigor mutant did not increase the duration of pauses in the dendrite shaft (5.1±0.8 s, *P*=0.9999, *n*=9; Fig. 4f). Similarly, the KIF1A rigor mutant had no effect on the percentage of spines invaded by syt-IV (3.0±0.9%, *P*=0.9821, *n*=12), the frequency (1.3±0.1, *P*=0.9973, *n*=9) or the dwell time of syt-IV puncta in spines (137.1±27.1 s, *P*=0.9452, *n*=19 invasions; Fig. 4h–j). These data indicate that sequestering KIF1A to the MTs by transfecting a KIF1A rigor mutant results in a markedly different phenotype than KIF1A knockdown, suggesting that KIF1A-kd releases syt-IV-containing vesicles from MTs in the dendrite shaft.

**Figure 5:**
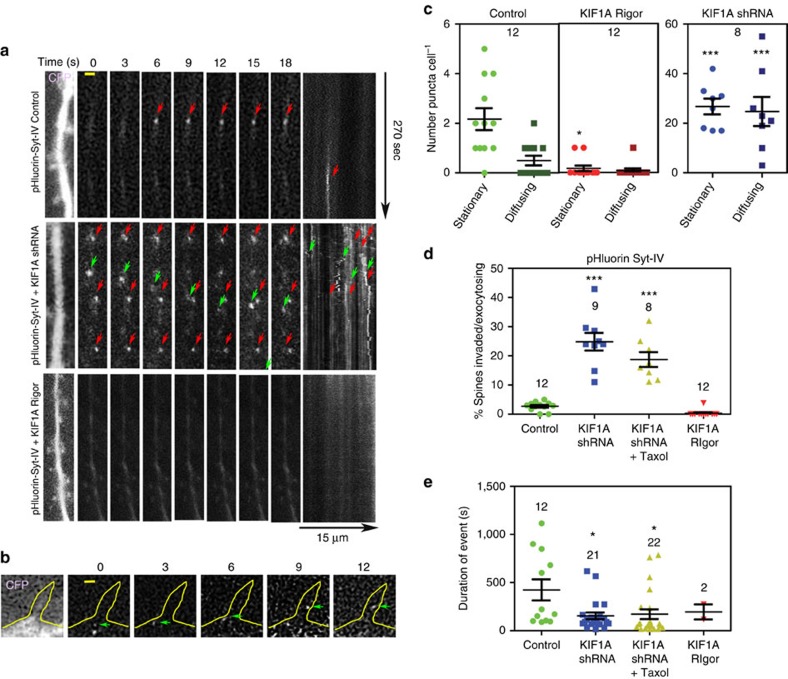
KIF1A knockdown causes more fusion and diffusion of syt-IV in the plasma membrane of dendrites and spines. (**a**) In control neurons, membrane-inserted pHluorin syt-IV puncta were rarely seen until an occasional exocytosis event (top panels), which typically remained stationary within the membrane during the time lapse (red arrows and kymograph). Neurons expressing KIF1A shRNA (middle panels) exhibited multiple stable membrane-inserted pHluorin-syt-IV puncta throughout the dendritic shaft (red arrows and kymograph) and a population of diffusing molecules within the plasma membrane (green arrows and kymograph). Cells expressing the KIF1A rigor mutant (bottom panels) rarely had syt-IV exocytotic events (scale bar, 1 μm). (**b**) Membrane-inserted syt-IV puncta were also observed to diffuse from the dendritic shaft into protrusions (green arrows, scale bar, 1 μm). (**c**) Cells expressing the KIF1A rigor mutant had a decrease in stationary membrane-inserted syt-IV puncta (*P*=0.0258). Conversely, cells expressing KIF1A shRNA had a marked increase in both stationary (*P*=0.0001) and diffusing (*P*=0.0001) puncta compared with controls. (**d**) The percentage of spines exhibiting exocytosis or invaded by membrane-inserted diffusing syt-IV increased significantly with the knockdown of KIF1A (*P*=0.0001). The addition of Taxol to cells expressing shRNA had no effect on the percentage of spines invaded by pHluorin syt-IV (*P*=0.1076) compared with shRNA KIF1A alone, but was significantly different than controls (*P*=0.0001). The KIF1A rigor mutant exhibited a ninefold trend towards a decrease in invasions as compared with control cells but was not significant (*P*=0.6907). (**e**) The duration (or dwell time) of pHluorin syt-IV puncta was significantly reduced in cells expressing KIF1A shRNA (*P*=0.0211) or cells with KIF1A shRNA plus Taxol (*P*=0.0340), as compared with controls, but were not significantly different from each other (*P*=0.9961). Asterisks indicate significance relative to controls (*P<0.05, **P<0.01, ***P<0.001). Numbers above scatter plots indicate the number of cells analysed from at least three replicate experiments. For all statistics, a one-way ANOVA with Tukey's *post hoc* test was used. All graphs show mean±s.e.m.

To determine whether the syt-IV-containing vesicles that are released from MTs can then fuse with the plasma membrane in the shaft or spines, we simultaneously expressed pHluorin-syt-IV and the KIF1A-kd construct. Interestingly, there was an order of magnitude more syt-IV exocytic events when KIF1A was knocked down (Fig. 5a,c). Moreover, we detected a marked increase in both stationary and diffusing populations (27.5±4.0 puncta cell^−1^ stationary and 25.2±7.2 puncta cell^−1^ diffusing, *P*=0.0001, *n*=8) as compared with controls (2.2±0.4 puncta cell^−1^ stationary and 0.50±0.11 puncta cell^−1^ diffusing, *n*=12; Fig. 5a,c). Conversely, when the pHluorin-syt-IV and KIF1A rigor mutants were simultaneously transfected, stationary exocytic events significantly decreased (0.17±0.11 puncta cell^−1^, *P*=0.0258, *n*=12) and diffusing pHluorin-syt-IV puncta trended towards a decrease (0.08±0.08, puncta cell^−1^, *P*=0.9999, *n*=12; Fig. 5a,c). There was also a prominent increase in the percentage of spines invaded by pHluorin-syt-IV after KIF1A knockdown, (24.8±3.0%, *P*=0.0001, *n*=9) but a decrease in the duration of these events (153.3±35.0 s, *P*=0.0211, *n*=21) as compared with the percentage of spines invaded (2.7±0.4%, *n*=12) and duration of events (423.6±109.7 s, *n*=12) in control cells (Fig. 5d,e), consistent with the increased diffusivity of the pHluorin-syt-IV puncta mentioned above (Fig. 5b). The increase in invasions (18.7±2.6%, *P*=0.0791, *n*=8) and decrease in duration of events (171.3±51.1 s, *P*=0.9993, *n*=22) were not affected by 10 nM Taxol addition (Fig. 5d,e), suggesting that MT dynamics did not play a role in the exocytosis of syt-IV. Expression of the KIF1A rigor mutant caused a trend towards a reduction in the percentage of spines invaded by pHluorin-syt-IV (0.37±0.32%, *P*=0.7264, *n*=12; Fig. 5d), but did not affect the duration of syt-IV pHluorin fluorescence (195.0±78.0 s, *P*=0.7151, *n*=2; Fig. 5e), compared with controls. Thus, causing KIF1A to stay attached to dendritic MTs but decreasing processivity resulted in a much different phenotype than decreasing the amount of KIF1A with shRNA. Together, these data indicate that syt-IV is not using MTs to enter spines after KIF1A knockdown. Rather, the results are consistent with increased exocytosis of syt-IV-containing vesicles throughout the dendritic arbor and diffusion into spines after KIF1A knockdown.

## Discussion

**Figure 6:**
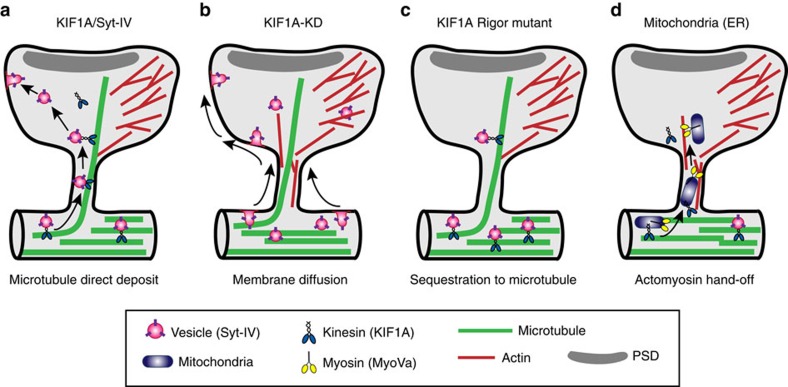
Working model of the direct deposit of cargo by MTs into spines. (**a**) Direct deposit model of delivering syt-IV-containing vesicles to the plasma membrane in spine heads, where KIF1A directly transports syt-IV into the spine head along newly polymerized invading MTs. (**b**) Knockdown of KIF1A causes dissociation from the MTs, unregulated fusion of syt-IV with the plasma membrane in both the shafts and spines, and diffusion of syt-IV within the membrane into spine heads. However, KIF1A knockdown does not disrupt MT polymerization into spines. (**c**) Expression of the KIF1A rigor mutant causes syt-IV to remain associated with MTs and leads to a decrease in membrane fusion and exocytosis. (**d**) Mitochondria enter dendritic spines via an acto-myosin mechanism independent of microtubules, as demonstrated by knockdown of MyoVa. MTs still invade these spines (not shown) but are not coordinated with mitochondria entry. Endoplasmic reticulum (ER) and recycling endosomes also enter spines via MyoVa and MyoVb (see refs 2,4,5).

Our data show that MTs, which polymerize into dendritic spines, provide a direct route for kinesin-based motor-driven transport of selective cargo into spines undergoing activity-dependent plasticity (Fig. 6a). Given that syt-IV-containing vesicles invade dendritic spines in a KIF1A-dependent manner, while mitochondria rarely invade spines along MTs, it is likely that these two cargos are being transported into the spines via distinct mechanisms. Indeed, our data with MyoVa knockdown suggest that mitochondria are using an actomyosin handoff (Fig. 6d). These data are also consistent with previous studies that show ER and recycling endosomes use MyoVa to enter spines^2^,^4^,^5^.

We also demonstrate that KIF1A is necessary for the maintenance of spines. Despite altered transport, the distribution of syt-IV puncta throughout the dendritic arbor was not significantly altered, suggesting that residual KIF1A was still capable of transporting syt-IV through the shaft, but that KIF1A knockdown and increased syt-IV entry into protrusions resulted in their reversion to filopodia-like protrusions. These data are consistent with syt-IV being a negative regulator of exocytosis^22^. In addition, these data further confirm that syt-IV is a specific cargo for KIF1A in the dendrites of hippocampal neurons. Our data also suggest that formation of a MT–motor–cargo complex (for example, MTs, KIF1A and syt-IV) in the dendrite shaft inhibits the vesicle and associated cargo from insertion in the plasma membrane until it is transported into spines along polymerizing MTs and released in the spine head (Fig. 6a). When KIF1A is knocked down, this MT–motor–cargo connection is disrupted and syt-IV-containing vesicles appear to enter spines in a manner more consistent with exocytosis in the dendrite shaft and spine, and diffusion in the plane of the membrane (Fig. 6b), even though MTs still enter spines in a manner similar to controls.

Moreover, our data suggest that motor proteins, in addition to their well-known function of transporting cargo throughout cells, also sequester that cargo to MTs while the motor is actively transporting the cargo. When KIF1A levels are decreased by shRNA knockdown, syt-IV-containing vesicles have fewer KIF1A motor proteins to bind and are therefore less likely to be associated with MTs in the dendrite shaft. Because the vesicles are not tethered to the MTs they can exocytose in the dendritic shaft and become very mobile, diffusing throughout the shaft and into spine heads (Fig. 6b), whereas the KIF1A rigor mutant keeps the vesicles tightly associated with the MTs, which results in a very different phenotype than the KIF1A-kd (Fig. 6c). In summary, we have discovered that motors are important for tethering vesicles and cargo; therefore, they can be delivered into specific locations, in this case to dendritic spines. When there are fewer motors present, the vesicles exocytose profusely along the dendrite shaft and become mobile in the plane of the plasma membrane.

Although our understanding of synaptic plasticity has increased greatly over the years, it is still unknown how the strength of certain synapses is increased during plasticity, while other synapses are unaffected. Nevertheless, targeted trafficking of materials in and out of specific spines is a key component of synaptic plasticity. Although MT invasions increase with neuronal activity^7^,^9^,^25^,^26^, it is unclear exactly what role MTs are playing, and, to date, no mechanism has been described that explains their contribution to plasticity. Here we provide direct evidence for a novel ‘direct-deposit' model^33^, where kinesin can directly transport cargos into the spine head along MTs that have transiently polymerized into spines from the dendrite shaft (Fig. 6a). This model opens up interesting possibilities for future studies and may provide insights into treatments of developmental and neurodegenerative disorders, which have demonstrated defects in spine physiology, such as Alzheimer's disease, Fragile-X syndrome and autism.

## Methods

### Plasmids and cloning

The rat eGFP-KIF1A was a kind gift from Gary Banker (OHSU). The tdTomato-KIF1A plasmid was constructed by restriction site removal of the eGFP from the eGFP-KIF1A plasmid and insertion of tdTomato. The pmTurquoise KIF1A shRNA vector was constructed by restriction site removal of eGFP from the commercially available pSuper vector (Oligoengine) and insertion of the cDNA coding for pmTurquoise (Addgene plasmid #36202). The mCherry-syt-IV and pHluorin-syt-IV were published elsewhere^22^,^30^. DsRed2-Mito was purchased from Clontech.

### Cell culture and transfections

Primary hippocampal neurons were prepared from Sprague Dawley rats (Harlan) at E18.5 (refs 34, 35). Briefly, rat hippocampi were dissected, trypsinized and plated at a density of 5 × 10^3^ neurons per cm^2^ on 1.0 mg ml^−1^ poly-D-lysine (Sigma)-coated coverslips, which were adhered to 35 mm plastic culture dishes containing a 15 mm hole drilled through the chamber. Neurons were plated with plating media (PM; neurobasal media with 5% fetal bovine serum (FBS), B27 supplement, 2 mM glutamine, 0.3% glucose and 37.5 mM NaCl) for 1 h at 5.0% CO_2_ and 37 °C after which the chambers were flooded with 2 ml of glial conditioned serum-free media (PM with no added FBS). A coverslip with a confluent glial feeder layer was placed above the neuronal cultures, and cultures were exchanged every other day with 700 μl of fresh serum-free media. Neurons were transfected at 9–12 DIV using the Lipofectamine 3000 reagent (Life Technologies) as per the manufacturer's instructions without the use of the P3000 supplemental reagent, which was toxic to our neuronal cell cultures. All procedures were approved by the University of Wisconsin Committee on Animal Care and were in accordance with the NIH guidelines.

### Live cell TIRF imaging

Images were acquired on a Nikon TE2000E microscope with total internal reflection fluorescence (TIRF) illuminator (Nikon), with a × 100/1.49 numerical aperture Plan Apo TIRF objective (Nikon), a Lumen Pro200 fluorescent illumination system consisting of a 200 W metal halide lamp and six-position excitation filter wheel and fibre optic illuminator (Prior Scientific), and a 10-position emission filter wheel. A Nikon perfect focus system was used for automatic focusing of the sample during time-lapse acquisition. GFP samples were excited using a 40 mW Argon 488 nm laser, and mCherry, DsRed and tdTomato were excited using a 10 mW solid-state 561 nm laser (Melles-Griot). Laser power was attenuated to 6.25% with neutral density filters. Images were recorded using an Evolve EMCCD or Coolsnap HQ-cooled interline charge-coupled device camera (Photometrics). For spine invasion data, quick successive time-lapse images were acquired in both the red and green channels at a rate of one frame 3 s^−1^ (0.33 hz) over a period of 20 min–1 h with a wide-field image of the mCerulean or pmTurquoise volume marker at the beginning and ending of each time lapse. For velocity data, images were acquired in a single channel at a rate of 5–10 frames s^−1^ (5–10 hz). During time-lapse microscopy, neurons were kept at 37 °C in a chamber enclosing the microscope and with a glass ring and sealed with silicone grease and a glass coverslip to maintain appropriate CO_2_ levels. For pharmacological treatments, cells were imaged for 5–20 min before bath application of 50 ng ml^−1^ BDNF (Sigma), 1 μm TTX (Enzo Life Sciences) or 10 nM Taxol (Sigma). Cells were incubated for 20 min followed by 20 min of imaging. For nifedipine treatment, 20 μm nifedipine (Sigma) was added to the cell cultures 24 h before imaging.

### Image analysis

Images were processed using MetaMorph (Molecular Devices) and/or ImageJ software (NIH). Dendritic spines were defined as protrusions <3 μm in length and having a head-width > neck-width, while filopodia were 3–10 μm in length with head-width ≤ the neck-width as previously described^36^. Spine invasions were determined by thresholding the raw images by 10 times the s.d. of the background and measuring the fluorescent intensity over time in a region of interest over spines above the threshold (Supplementary Fig. 8). Only fluorescence intensities above the designated threshold were counted as a spine invasion. The % of spines invaded was determined by dividing the number of invaded spines over a 20-min period by the total number of spines in the dendritic field. Invasion frequency was defined by the total number of invasions divided by the number of spines invaded. Dwell times were defined as the duration of time in which a given event persisted in a spine, and data were only included for events that began and ended within the duration of the time lapse. Instantaneous velocities were calculated by the slope of the line produced by the kymograph tool in ImageJ and did not include stationary or paused events. Fluorescence intensity measurements were performed using wide-field microscopy, and the average background was subtracted from the entire image. Normalized fluorescence was measured in three to five regions of interest along the dendrites of a given cell. All images for pHluorin intensity measurements were corrected for photobleaching before measuring the fluorescence intensity of pHluorin over time.

EB3 comets were imaged at 0.33 Hz and analysed using the ImageJ plugin MTracksJ. Velocites, lifetimes and track lengths were calculated using 200–400 EB3 comets per cell. For the MSD analysis^37^,^38^, images were captured at 5–10 hz for 100 s and syt-IV puncta were tracked overtime using the image J plugin MTrackJ. The general expression for the MSD is given by:


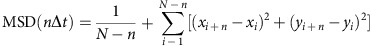


Where *N* is the total number of frames in the trajectory, *n* is the number of frames for different time intervals, Δ*t* is the time between frames and *x*_*i*_ and *y*_*i*_ are the positions of syt-iv in frame *i* (refs 37, 38, 39). The diffusion constant (*D*) was estimated from the slope of the MSD verses Δ*t* when plotted on a linear axis. When the MSD is plotted on a log–log axis, it provides insight into the type of motion being observed^39^. For various types of motion the relationship between the MSD and time interval (*n*Δ*t*) can be generalized to MSD ∼(*n*Δ*t*)^*α*^, where *α* is the diffusive exponent^39^. For randomly diffusing particles, *α*=1 and for directed motion at a constant velocity, *α*=2. By plotting the MSD verses Δ*t* on the log–log axis, *α* is the slope of the MSD.

### Rat KIF1A and MyoVa shRNA and rescue experiments

A previously published shRNA sequence^14^ directed against rat KIF1A was cloned into the pSuper shRNA vector system (Oligoengine) and transfected in primary rat hippocampal neurons. The 62mer sequence is as follows: 5′-GATCCCCTACCTATGTGAACGGCAAGTTCAAGAGACTTGCCGTTCACATAGGTATTTTTA-3′ with the shRNA sequence and reverse compliment underlined and separated by a nine-base pair linker. The shRNA-resistant mutant was created by the incorporation of five point mutations in the targeted shRNA region of the rat eGFP-KIF1A plasmid, which change the DNA sequence without effecting the amino-acid composition of the translated protein. The point mutations were performed using the QuikChange lightning multi site-directed mutagenesis kit (Agilent) and are as follows: 5′-CACTTACGTGAATGGCAAA-3′ (T1692C, C1695T, T1698C, C1704T, G1710A). For MyoVa knockdown the following 63mer sequence was used: 5′-GGTCTCTGTTTCATTTATCTTCAAGAGAGATAAATGAAACAGAGACC-3′. Efficiency of the knockdown of endogenous MyoVa, KIF1A, and the rat eGFP-KIF1A and eGFP-KIF1A shRNA-resistant mutant constructs were determined by immunocytochemistry and/or western blot analysis.

### Western blotting and immunocytochemistry

For KIF1A western blots, HEK cells (American Type Culture Collection) were grown to confluency in a 75 cm^2^ flask, trypsinized, pelleted at 1,000 r.p.m. for 3 min and transfected using Lipofectamine 3000 (Invitrogen) with rat eGFP-KIF1A, the rat eGFP-KIF1A shRNA-resistant mutant, KIF1A shRNA plus rat eGFP-KIF1A or KIF1A shRNA plus the shRNA-resistant eGFP-KIF1A mutant. For MyoVa western blots, primary E18.5 rat hippocampal neurons were transfected by nucleofection with a transfection efficiency of 40–60%. The transfected neurons were then plated and the shRNA was expressed for 72 h before cell lysis. The total protein concentration of lysates was determined using the BCA protein assay (Thermo Scientific), and equimolar amounts of protein were separated using SDS–PAGE and transferred to a polyvinylidene fluoride membrane. Blots were probed with primary rabbit anti-KIF1A (1:5,000; AbCam) or primary rabbit anti-MyoVa antibodies (Thermo Fisher) and secondary horseradish peroxidase anti-rabbit antibodies (1:5,000; Thermo Scientific). Western blots were viewed using a ChemiDoc-It imaging system (Bio-Rad). For full western blots, see Supplementary Fig. 9.

For immunocytochemistry, neurons were fixed in 4% paraformaldehyde-KREB-sucrose at 37 °C, rinsed in PBS, blocked with 10% BSA and permeabilized in 0.2% Triton X-100 (ref. 34). Neurons were incubated for 1 h at room temperature with primary rabbit anti-KIF1A (1:500; Abcam) antibodies, followed by a 1 h incubation with the secondary goat anti-Rabbit alexa-568-conjugated antibodies (1:500; Life Technologies). Fixed neurons were imaged in wide field, and fluorescence intensities of KIF1A were compared between neurons transfected with the rat KIF1A shRNA and neighbouring untransfected neurons, or neurons transfected with the shRNA control vector.

### Graphing and statistics

Graphpad Prism was used for all graphing and statistical analyses. For all data comparing more than two conditions, a one-way analysis of variance and Tukey's *post hoc* tests were performed. For data comparing only two conditions, a Student's *t*-test was performed. Data with *P* values less than 0.05 were considered statistically significant. On all graphs *P<0.05, **P<0.01 and ***P<0.001.

### Data availability

The authors declare that the data supporting the findings of this study are available within the article and its Supplementary Information Files.

## Additional information

**How to cite this article:** McVicker, D. P. *et al.* Transport of a kinesin-cargo pair along microtubules into dendritic spines undergoing synaptic plasticity. *Nat. Commun.* 7:12741 doi: 10.1038/ncomms12741 (2016).

## Supplementary Material

Supplementary Information Supplementary Figures 1-9
