# Supplementary material for: Transport of a kinesin-cargo pair along microtubules into dendritic spines undergoing synaptic plasticity
Source: Nat Commun. 2016 Sep 23;7:12741. doi: 10.1038/ncomms12741 (PMC5411814; doi:10.1038/ncomms12741)
Supplement: Supplementary Information — Supplementary Figures 1-9 [file ncomms12741-s1.pdf]

Supplementary Figures and Legends

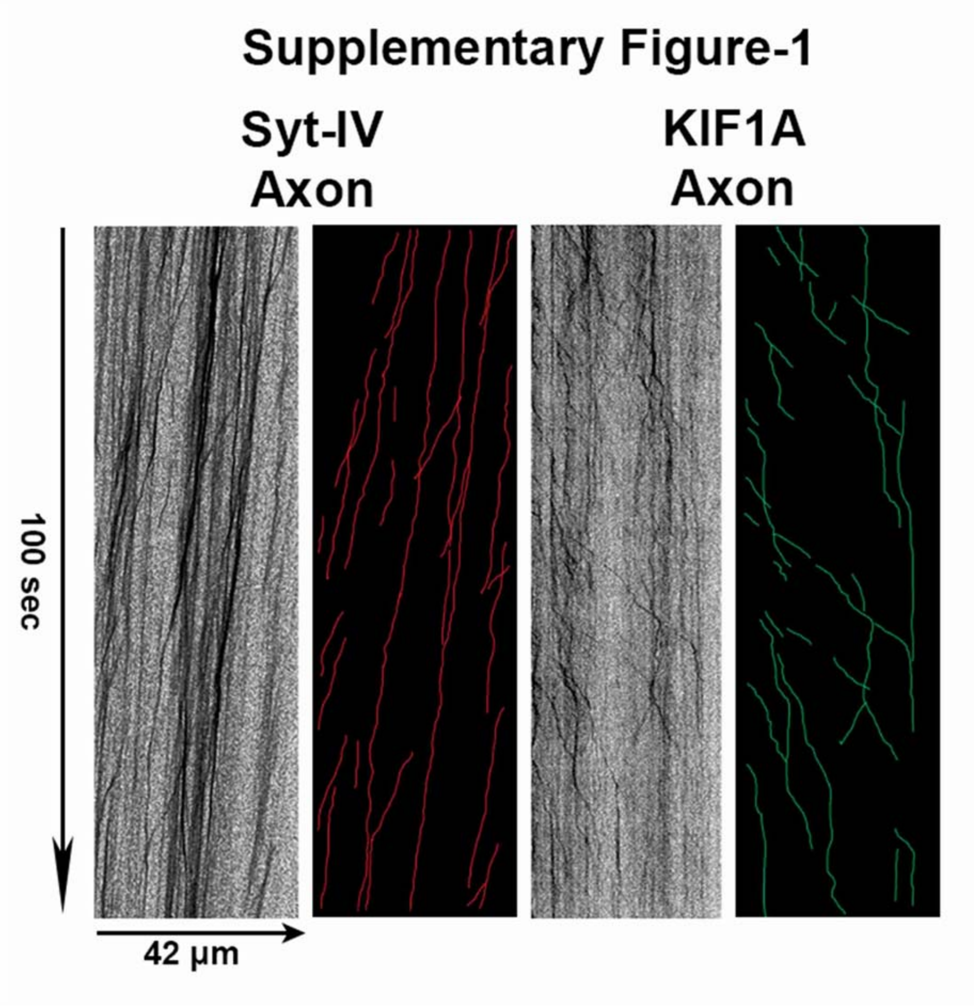

Supplementary Figure 1 KIF1A and syt-IV move in opposing directions in axons. Kymographs were made of eGFP-KIF1A and mCherry-syt-IV movement in axons from time-lapse sessions collected at 5-10 Hz for 100 seconds. KIF1A moves primarily in the anterograde direction (selected traces in green), while syt-IV moves retrogradely (selected traces in red) toward the neuron cell body. These data indicate that, unlike in the dendrites (Fig. 1), KIF1A and syt-IV do not co-localize and move together in axons.

Supplementary Figure-2

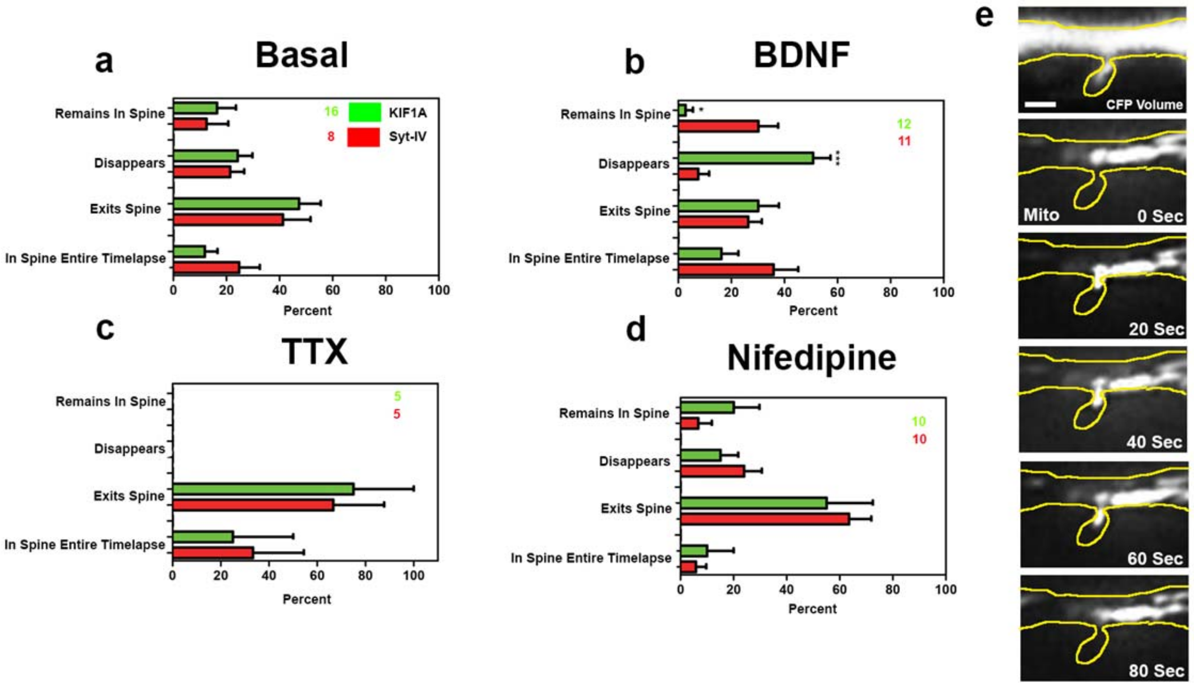

Supplementary Figure 2 Behavior of KIF1A puncta, syt-IV puncta and mitochondria in dendritic spines. (a) At basal levels KIF1A and syt-IV demonstrated similar behaviors in dendritic spines and remained in the spine for the duration of the time lapse after the initial invasion 21.9 ± 10.8% and 12.5 ± 8.2% of the time while disappearing from the spine 28.5 ± 8.5% and 21.4 ± 5.3% of the time, respectively. KIF1A and syt-IV exited the spine 50.2 ± 11.2% and 41.3 ± 10.4% of the time, while 12.8 ± 5.3% and 24.8 ± 7.8% of KIF1A and syt-IV puncta were in the spines prior to the start of imaging and remained in the spine during the entire time lapse (n=14). **(b)** After a 20-minute bath application of BDNF, the percentage of KIF1A that remained in the spine after invasion was significantly less (2.8 ± 2.7%, p = 0.0306) than was seen for syt-IV (30.2 ± 7.5%) and the percentage of KIF1A puncta that disappeared was significantly more (50.8 ± 6.6%, p = 0.0011) than syt-IV (6.9 ± 3.8%). There was no statistical difference between the percentages of puncta observed to exit the spine (30.2 ± 7.7% for KIF1A and 26.4 ± 5.1% for syt-IV p = 0.9999) or that were in the spine the entire duration of the time lapse (16.3 ± 6.4% for KIF1A and 35.9 ± 9.3% for syt-IV, p = 0.6008, n= 12). **(C)** No KIF1A or syt-IV puncta were observed to remain in the spine after the initial invasion or disappear with a 20-minute bath application of TTX. However, a large percentage of KIF1A (75.0 ± 25.0%) and syt-IV puncta (66.7 ± 21.1%) exited the spine after invading, while a smaller percentage was in the spines the entire duration of the time lapse (25.0 ± 25.0% for KIF1A and 33.3 ± 21.1% for syt-IV, n=6). **(d)** KIF1A and syt-IV behaved similarly after 24-hour nifedipine treatment with the majority of events exiting the spines (55.1 ± 17.4% for KIF1A and 63.6 ± 8.3% for syt-IV; p = 0.7615) and a lesser percent remaining in the spines (20.1 ± 9.7% for KIF1A and 6.7 ± 5.1% for syt-IV; p = 0.9999), disappearing (15.1 ± 6.7% for KIF1A and 24.0 ± 6.7% for syt-IV; p = 0.9999), or in the spine the entire time lapse (10.0 ± 6.7% for KIF1A and 5.8 ± 4.0% for syt-IV, p = 0.9999, n= 10). **(e)** An example time-lapse series showing that mitochondria do not penetrate deep into the spine head. Scale bar, 2µm. A one-way analysis of variance with Tukey's *post-hoc* was used. Data reported as mean ± s.e.m. Asterisks above data points indicate significance relative to controls.

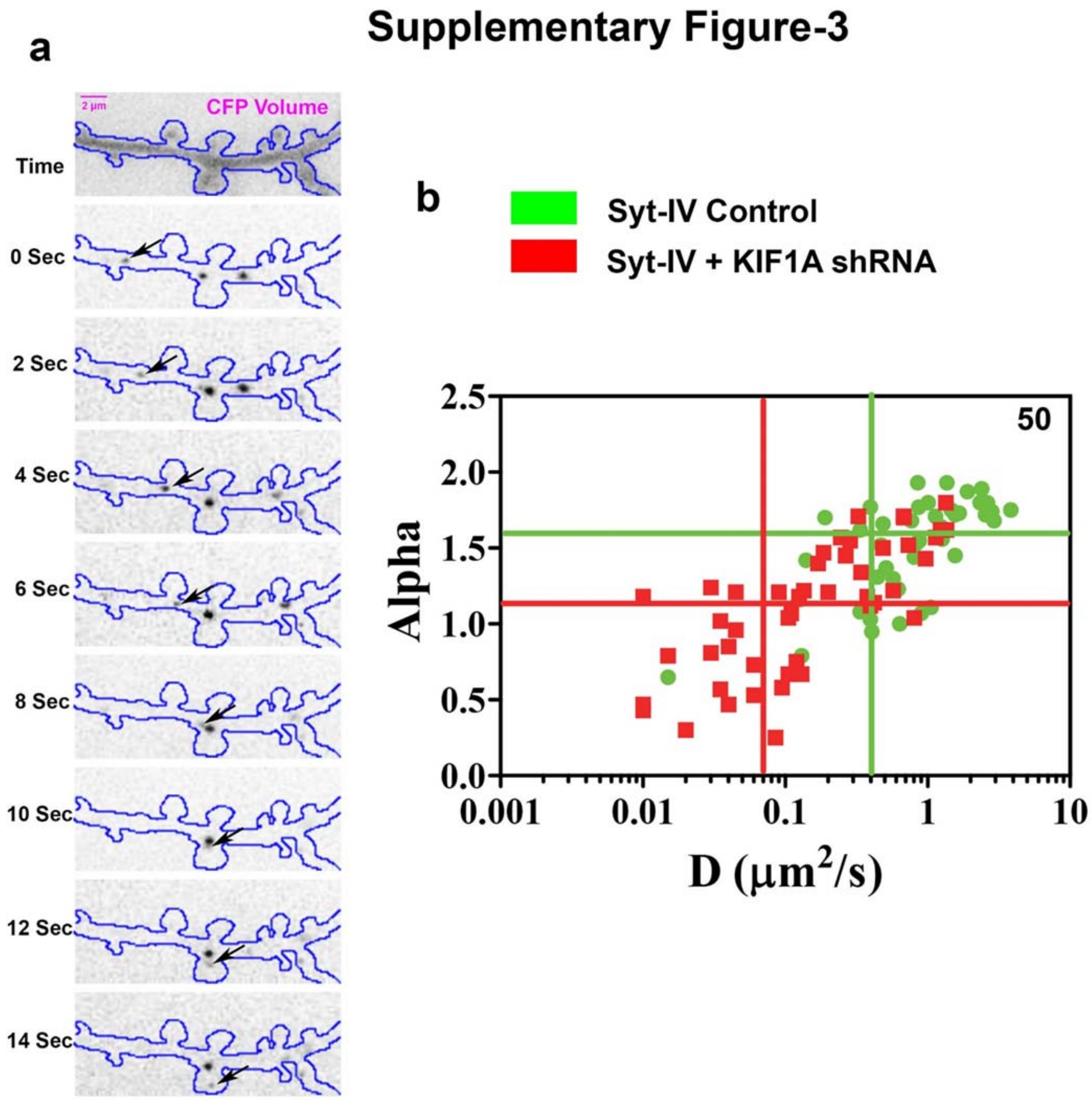

Supplemental Figure 3 Syt-IV moves in the dendritic shaft and into spines in a directed motion. (a) Images showing a syt-IV punctum (arrows) processively moving along a stretch of dendrite into a dendritic spine (scale bar 2 µm). **(b)** Mean square displacement (MSD) analysis of syt-IV in control neurons (green) and syt-IV in cells expressing KIF1A shRNA (red). Syt-IV puncta in neurons expressing KIF1A shRNA have a significantly reduced diffusion coefficient (from 1.1 ± 0.05 $\mu m^2/s$ in controls to 0.3 ± 0.13 $\mu m^2/s$ in shRNA expressing cells, p = 0.0001, n= 50 tracks) and diffusive exponent, α, (from 1.6 ± 0.06 in controls to 1.1 ± 0.05 in neurons expressing KIF1A shRNA, P = 0.0001, n =50 tracks) compared to controls. These data indicate that syt-IV puncta move in a predominantly directed manner with a constant velocity in control cells, but movement of some populations becomes more consistent with random diffusion in cells expressing shRNA. A student's t-test was used to determine significance. Data reported as mean ± s.e.m.

Supplementary Figure-4

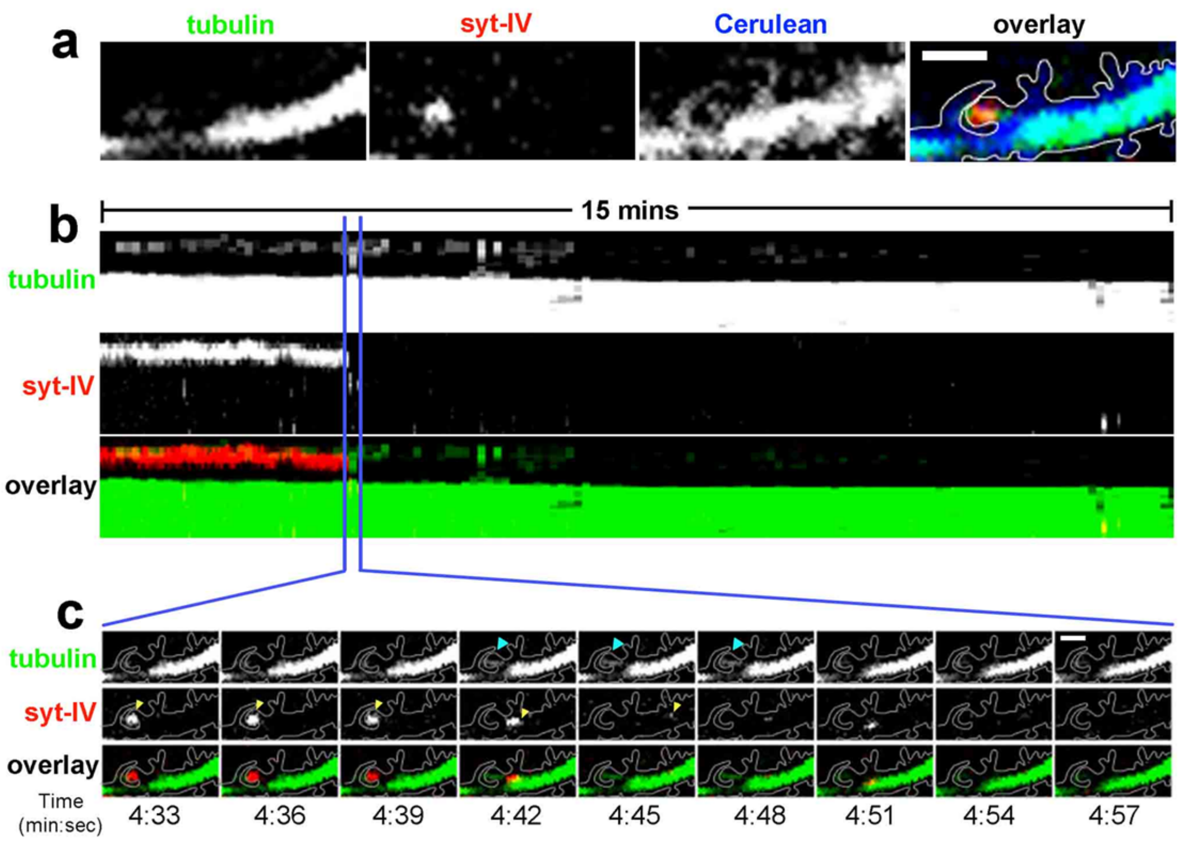

Supplementary Figure 4 Syt-IV exits dendritic spines after MT invasions. (a) Images showing tubulin, syt-IV, and a CFP volume fill (scale bar 2 μm). **(b)** Kymograph of a MT entering a dendritic spine followed by the exit of syt-IV from the same spine. **(c)** Time-lapse images demonstrating a MT invasion of a dendritic spine (cyan arrows) followed by the exit of a syt-IV puncta along the MT track (yellow arrows).

Supplementary Figure-5

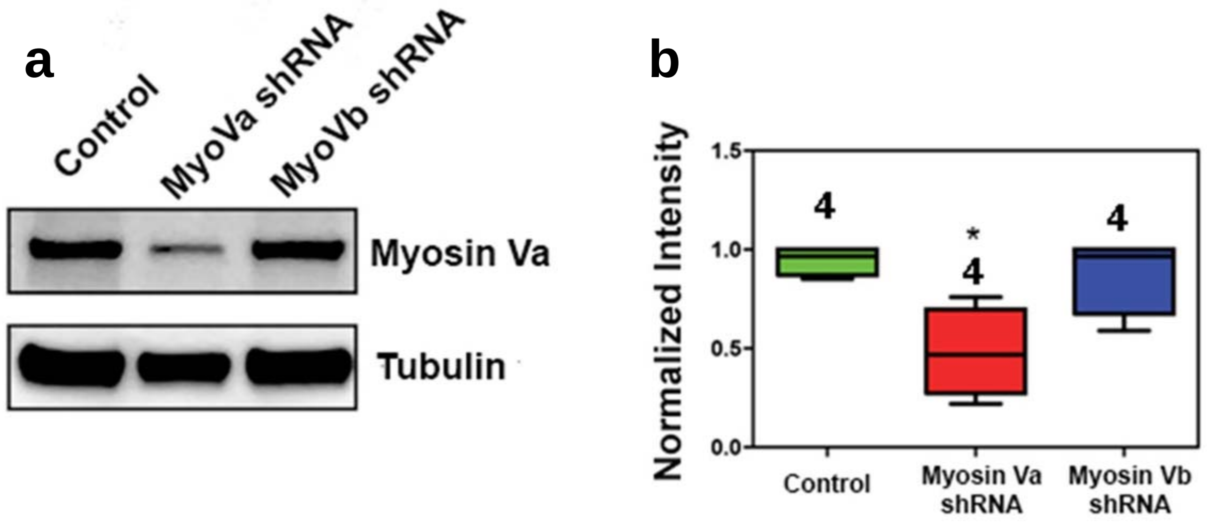

Supplementary Figure 5 shRNA mediated knock down of myosin Va. (a) Western blot demonstrating the knockdown of myosin Va by shRNA. E18.5 primary rat hippocampal neurons were transfected with the shRNA plasmid, control plasmids, or myosin Vb shRNA plasmids by nucleofection, with a transfection efficiency between 40%-60%, and harvested 72 hours after transfection. **(b)** Quantification of the Western blot demonstrates that the normalized intensity of myosin Va bands was significantly reduced in cells expressing the myosin Va shRNA (0.5 ± 0.1 AU, p =0.0166 n = 4) compared to cells transfected with the control plasmid (0.950.04AU) or myosin Vb shRNA (0.9 ± 0.2AU). Given the low transfection efficiency, these numbers are an underestimate of the actual knock down efficiency. A one-way analysis of variance with Tukey's *post-hoc* was used. Data reported as mean ± s.e.m. Asterisks above data points indicate significance relative to controls.

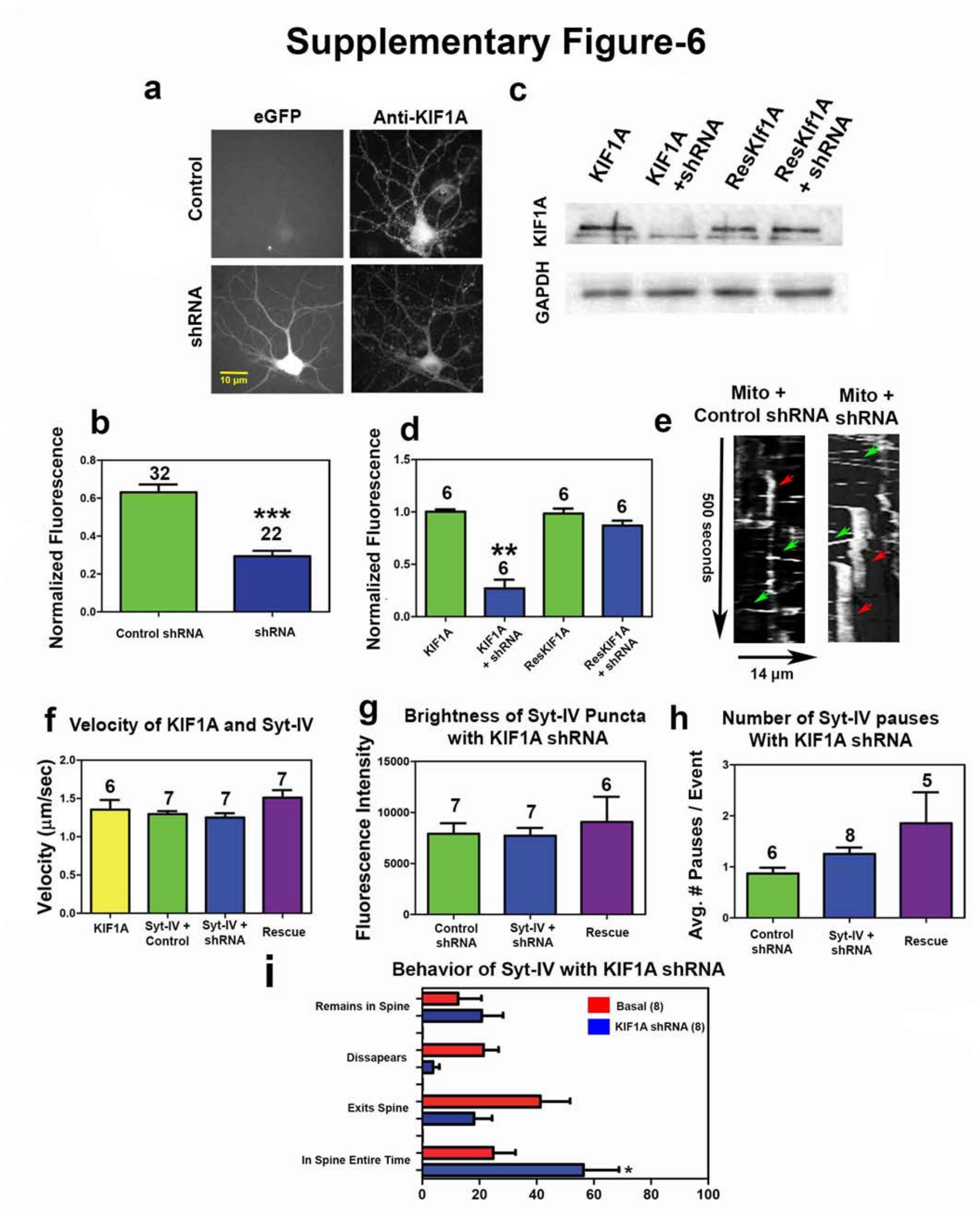

Supplementary Figure 6 shRNA mediated knockdown of KIF1A does not cause changes in mitochondria or velocity, brightness and number of syt-IV pauses in dendrites. (a) Immunocytochemistry was used to measure the fluorescence intensity of endogenous KIF1A in control neurons (top) and neurons expressing the eGFP-pSuper construct containing shRNA against KIF1A (bottom; scale bar is 10 μm). **(b)** The normalized fluorescence intensity of KIF1A in neurons expressing KIF1A shRNA was significantly less (0.29 ± 0.03, p = 0.0001, n= 22) than neurons not expressing the shRNA construct (0.63 ± 0.04, n= 32). **(c)** Western blotting was also used to quantify the efficiency of KIF1A knockdown by expressing the KIF1A construct and the

shRNA-resistant mutant construct (ResKIF1A) mutant in HEK293 cells, with or without the KIF1A shRNA construct. **(d)** The normalized intensity of the bands in Western blots were measured in ImageJ, and cells expressing both the KIF1A construct and shRNA were of significantly lower intensity (0.27 ± 0.08, p = 0.0014, n= 6) than those of either KIF1A alone (1.00 ± 0.02, n=6), the rescue mutant alone (0.99 ± 0.05, n= 6), or the rescue mutant with shRNA (0.87 ± 0.12, n= 6). **(e)** Kymographs of mitochondria moving through the dendritic shaft of control neurons (left) and neurons expressing KIF1A shRNA (right). In controls and shRNA expressing neurons mitochondria moved in a similar manner having both stationary (red arrows) and motile (green arrows) events in approximately the same ratios and moving at similar velocities between conditions. **(f)** KIF1A shRNA had no significant effect on the velocity of syt-IV puncta (1.3 ± 0.1 µm/s, p = 0.7809, n= 7) as compared to control neurons (1.3 ± 0.04 µm/s, n= 7), which were also similar to the velocity of KIF1A puncta (1.3 ± 0.1 µm/s, p = 0.9382, n= 6). The velocity of syt-IV puncta was also statistically similar to controls when expressing both KIF1A shRNA and the KIF1A rescue construct (1.5 ± 0.1 µm/s, p = 0.9401, n= 7). **(g)** The brightness (7907 ± 1040 fluorescent units for control neurons, 7720 ± 766 fluorescent units for shRNA neurons, and 9071 ± 2455 fluorescent units for rescue neurons, p = 0.9050 and p = 0.3952, n= 7, 7, and 6, respectively) and **(h)** average number of pauses per event (0.87 ± 0.11 pauses/event for control neurons, 1.3 ± 0.1, p = 0.9999, pauses/event for shRNA neurons, and 1.9 ± 0.6 pauses/event for rescue neurons, p = 0.9999, n= 6, 8, and 5, respectively) of syt-IV puncta moving along the dendritic shaft were also unaffected by the expression of KIF1A shRNA and were statistically similar to control and rescued neurons. **(i)** There was a higher percentage of Syt-IV puncta in spines the entire time lapse with KIF1A shRNA expression (blue bars) as compared to control neurons (red bars), from 24.8 ± 7.8% in control neurons to 56.4 ± 9.7% (p = 0.0483, n= 8). Asterisks above data points indicate significance relative to controls. For panel **b,** a student's t-test was used to determine significance. For panels **d, f, g, h,** and **i,** a one-way analysis of variance with Tukey's *post-hoc* test was used. All graphs show mean ± s.e.m.

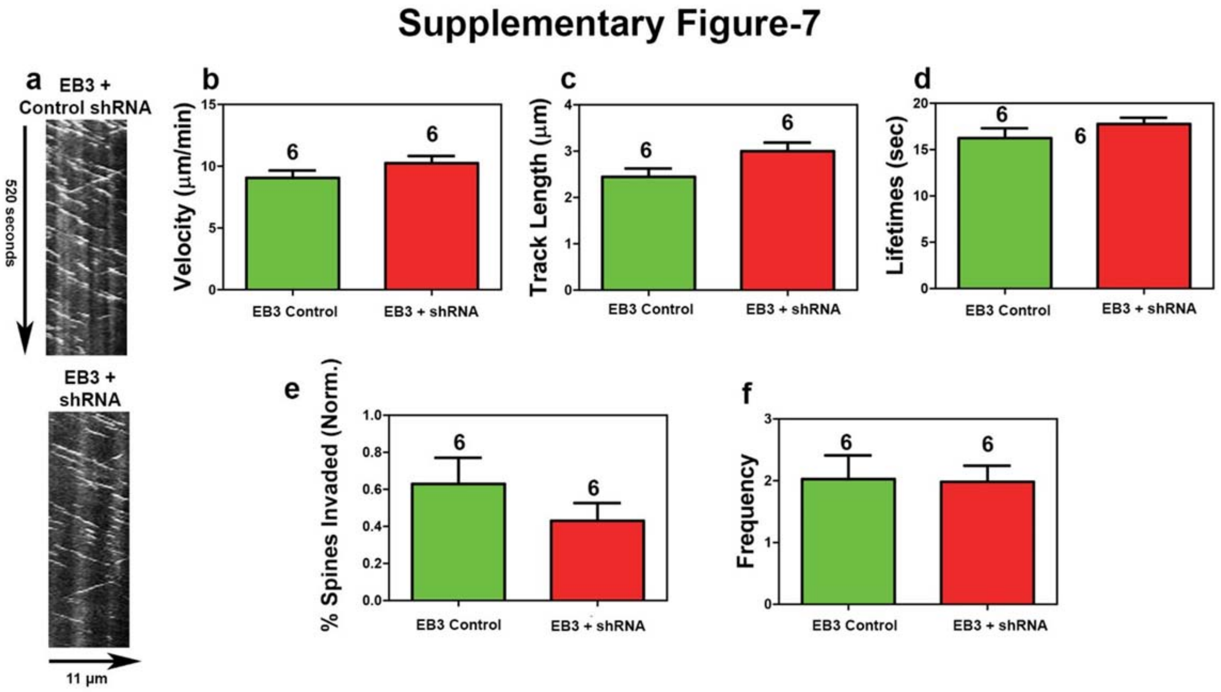

Supplementary Figure 7 KIF1A knock down does not affect MT dynamics in the dendritic shaft, or the rate and frequency of MT spine invasions. (a) Kymographs of the movement of the MT plus end binding protein EB3 through the dendritic shaft. **(b)** Velocities of EB3 comets in neurons expressing KIF1A shRNA (10.2 ± 0.6 µm/s, p = 0.4149, n= 6) were statistically similar to those in control neurons (9.1 ± 0.6 µm/s, n=6). **(c)** KIF1A shRNA also had no effect on the EB3 track length (2.4 ± 0.2 µm for control neurons and 3.0 ± 0.2 µm for shRNA expressing neurons, p = 0.0792, n= 6), **(d)** or the lifetimes of the EB3 comets (16.2 ± 1.1s for control neurons and 17.8 ± 0.7s for shRNA expressing neurons, p = 0.0852, n= 6). Approximately 200-300 EB3 comets were analyzed for each neuron (n value). **(e)** No statistically significant differences were observed in the percentage of spines invaded by EB3 (p = 0.5556). The normalized percentage of spines invaded by EB3 was 0.6 ± 0.1% for control neurons and 0.4 ± 0.1% for neurons expressing shRNA (n= 6). **(f)** Invasion frequencies of EB3 were likewise unaffected by KIF1A shRNA with frequencies for control neurons being 2.0 ± 0.4 and 2.0 ± 0.3 for shRNA expressing neurons (p= 0.9284). A student's t-test was used to determine significance. All graphs show mean ± s.e.m.

Supplementary Figure-8

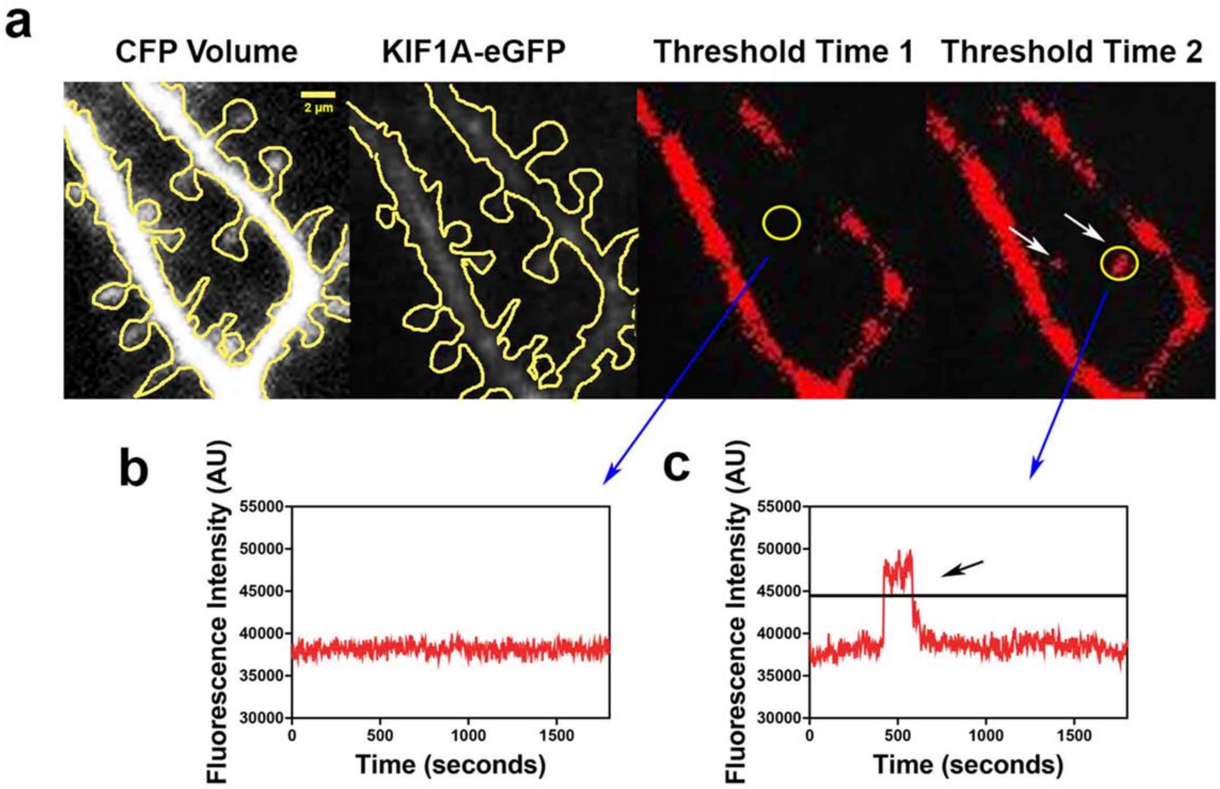

Supplementary Figure 8 Analysis of spine invasions using thresholding. (a) Example of a neuron expressing eGFP-KIF1A before thresholding (panel two), after thresholding but prior to an exocytosis event (panel three), and after two exocytosis events (white arrows panel four). The yellow circle in panel three is a ROI measuring background fluorescence and corresponds to the fluorescence intensity measurement in (scale bar is 2 µm) **(b)**, while the yellow circle in panel four is a ROI measuring the fluorescence of an exocytosis event corresponding to the measurements in **(c).** The threshold was set to ten standard deviations above the background (black line in c).

Supplementary Figure-9

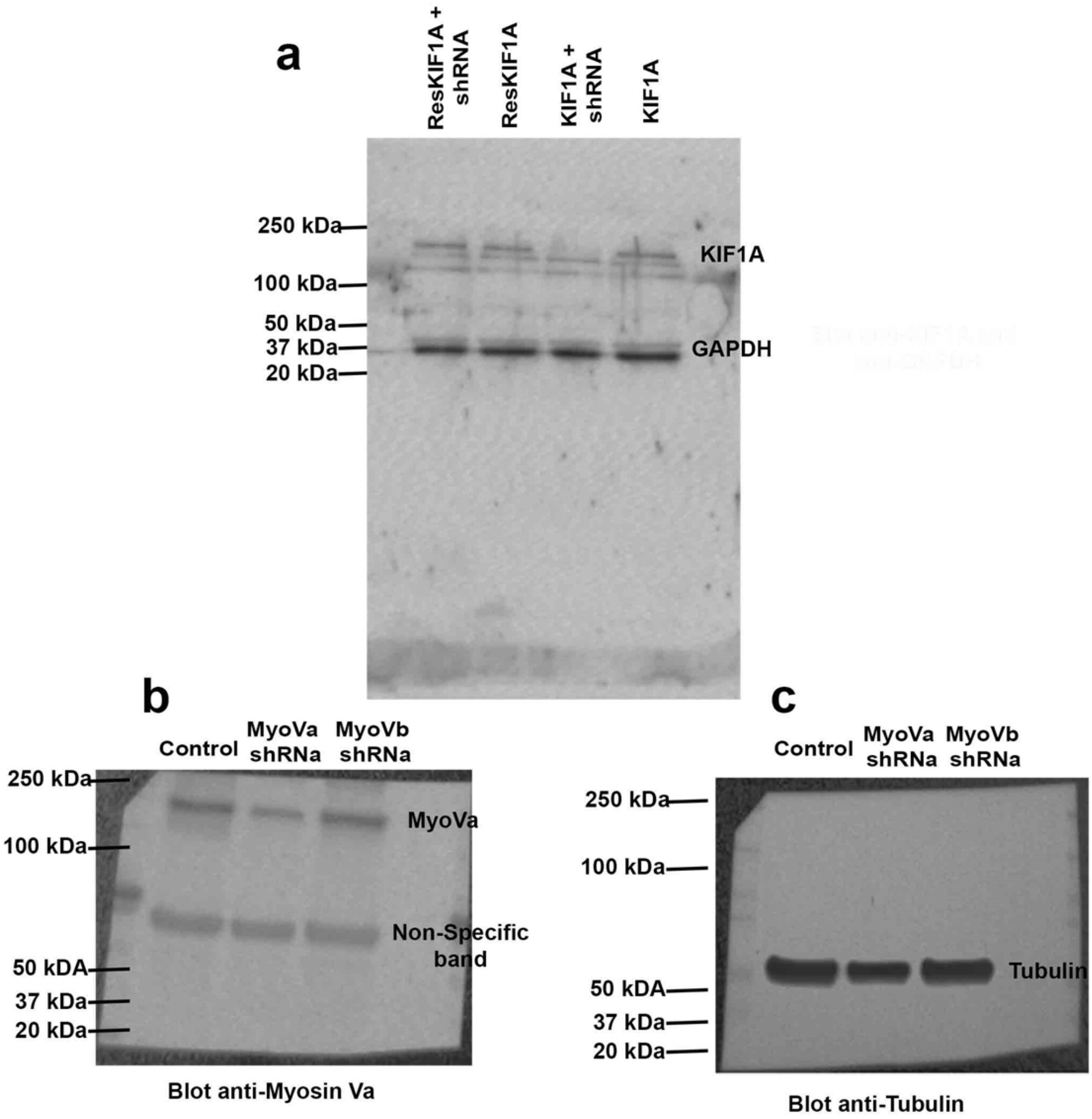

Supplementary Figure 9 full KIF1A and Myosin Va western blots. (a) Full Western blot of KIF1A expressed in HEK cells with KIF1A + shRNA, KIF1A recue, and KIF1A rescue + shRNA. Westerns were co-stained with GAPDH as a loading control. **(b)** Full Western blot from rat hippocampal cells nucleofected with control plasmids, myosin Va shRNA, or myosin Vb shRNA as an additional control. **(c)** The Western in panel **b** was stripped and re-blotted for α-tubulin for a loading control.
